# Assessing Genetic Diversity and Population Structure of Western Honey Bees in the Czech Republic Using 22 Microsatellite Loci

**DOI:** 10.3390/insects16010055

**Published:** 2025-01-09

**Authors:** Aleš Knoll, Martin Šotek, Jan Prouza, Lucie Langová, Antonín Přidal, Tomáš Urban

**Affiliations:** 1 Department of Animal Morphology, Physiology and Genetics, Faculty of AgriSciences, Mendel University in Brno, Zemědělská 1, 613 00 Brno, Czech Republic; ales.knoll@mendelu.cz (A.K.);; 2 Department of Animal Breeding, Faculty of AgriSciences, Mendel University in Brno, Zemědělská 1, 613 00 Brno, Czech Republic

**Keywords:** *Apis mellifera*, population genetics, Central Europe, sampling method, genetic group, district

## Abstract

To date, no study has been conducted to investigate the diversity in honeybee populations of *Apis mellifera* in the Czech Republic. Between 2022 and 2023, worker bees were collected from colonies distributed throughout the Czech Republic in 77 districts, and their genetic differences were examined using 22 microsatellite loci. The samples were obtained from hives (*n* = 3647) and through the process of capture on flowers (*n* = 553). Genetic diversity parameters were assessed for both populations in all 77 districts. The findings demonstrated that honeybee populations exhibit moderate genetic diversity, as evidenced by the number of observed alleles, the Shannon index, and heterozygosity values. There was no discrepancy in diversity between hive and flower samples. Diversity characteristics were determined: mean observed heterozygosity 0.55 (hives) and 0.56 (flowers), and fixation index 0.58 for both populations. The average number of alleles per locus was 13.77 and 11.18 from hives and flowers, respectively. The low *F_ST_* and *F_IS_* values (they measured the level of genetic differentiation between populations and the level of inbreeding, respectively) suggest the absence or minimal genetic diversity within and among studied populations. The genetic variation was calculated as 2% and 1% between populations, 8% and 6% between individuals within populations, and 91% and 93% between all individuals in samples from hives and flowers, respectively. Cluster and DAPC (discriminant analysis principal component) analysis classified the bee samples collected from across the country into three and five to six distinguishable groups, respectively. The honeybee population in the Czech Republic displays sufficient diversity and a partial structure. However, there appears to be no correlation between the genetic groups and the geographic regions to which they are assigned.

## Simple Summary

The present study employed a total of twenty-two microsatellite loci for the purpose of assessing and analyzing the genetic diversity between and within different populations of honeybees in the Czech Republic. A total of 3647 samples of bees from hives and 553 samples from flowers were collected uniformly from 77 geographical regions (districts). All investigated microsatellite markers demonstrated sufficient variability and moderate heterozygosity. Additionally, the population structure was identified. The findings of this study indicate that there is moderate genetic diversity among honeybee populations defined by districts, but this is independent of evidence for at least three major genetic clusters.

## 1. Introduction

The Czech Republic registers about nine honeybee colonies per km^2^ [1], which is one of the highest colony densities all over the world [2,3]. There are about eight beekeepers registered per 10 km^2^ (65,058 in total) and 162 inhabitants per beekeeper, which are the highest and the lowest values, respectively, in the world [3]. Moravia is a territory with distinctly higher colony density in comparison with Bohemia [4]. Density is in some Czech regions higher than in others, and the colony distribution is not even. In some land registries, the colony density is under five colonies per km^2^ or up to zero (e.g., Šumava or Ore Mountains), and, on the other hand, some of them are over-crowded with more than 50 colonies per km^2^ [5]. Such high colony density is undesirable in relation to the spread of bee diseases, especially varroosis [6]. The colony collapse disorder is a frequent phenomenon in many apiaries and every year [5]. The mean number of colonies per beekeeper is about 11 colonies in the last 20 years, and only under 140 beekeepers keep over 150 colonies [1]. Thus, it distinctly prevails as a hobby rather than professional beekeeping in Czechia. The number of breeding stations decreased during 2015–2020 from 76 to 46 breeders. Consistently, this trend was decreasing the number of reared honeybee breeding queens from 45 to 30 thousand queens per year [1]. Thus, most queens are currently reared at their own apiary.

In most of the territory of today’s Czechia, the native race was the European Dark honey bee, *Apis mellifera mellifera* Linnaeus, 1758, [7,8] with the exception of the southern region of Moravia where the Carniolan race (*A. m. carnica* Pollmann 1879) natively reached [9,10,11]. Natural/accidental or intentional crossing of the native race with other imported races on the Czech territory is well documented from the middle of the 19th century at the latest [12]. In particular, the following races are predominantly mentioned: Carniolan, Italian (*A. m. ligustica* Spinola 1806), Caucasian (*A. m. caucasia* Pollmann 1889), Anatolian (*A. m. anatoliaca* Maa 1953), Macedonian (*A. m. macedonica* Ruttner 1988) and the Cyprian bee (*A. m. cypria* Pollmann 1889). The beginning of bee race hybridization in today’s Czech territory is not possible to specify. The first mentions of interest in the Carniolan race are already documented from the period of Anton Janscha, about 1750, and later also an imperial teacher in Viennese “*Theresianische Imkerschule*” [8,13]. The above-mentioned crossings resulted in negative breeding experiences followed by a criticism emphasizing that adaptation to the local environment is suitable for the selection and breeding of the honeybee [14]. This criticism is consistent with current scientific findings [15]. However, the influence of non-native races continued. In 1949, Tomšík [10] found that Czech honeybees showed morphological characters of both Carniolan and Dark races, and he named the Czech honey bee population “a Central European Honey Bee”, which probably corresponds to the statement by Ruttner [8] about “Brown European bee”. Subsequently, Veselý [11] found with using of morphometric characters that most of the Czech Regional breeding stations already kept Carniolan race. Only in West Bohemia (Tachov), he found morphometric characters close to the Dark Bee but “not in a pure form”. He noted that this finding cannot confirm the presence of any Dark bee population in this subregion.

The period of efforts to improve honeybee breeding, but without a firmer framework, after the Second World War [16] was followed by a decision [11] (already just formal under these circumstances at that time) to use a crossing method, grading-up, to replace remaining traces of the Dark race by the Carniolan race imported from Austria. This formal decision was based on the results of the test with 120 Troiseck queens from Austria. The imported queens showed insignificantly higher honey production and significantly more favorable temperament and calmness. Thus, the suggestion about the necessity to select and breed primarily a local bee [14,15] was not followed. A similar development is described by Wragg et al. [17] in France, where honeybees of C lineage were/are preferred due to breeder-friendly properties, and it resulted in varying degrees of admixture and a significant contribution of C lineage alleles to the endogenous Dark bee. De la Rúa et al. [18] stated that the intense dissemination of Italian and Carniolan honey bees throughout the European continent has resulted in the almost complete replacement of the Dark bee by the Carniolan bee in Central European countries such as Germany and the hybridization of all three subspecies in Scandinavian countries and the British Isles. That is why activities for the conservation of the Dark bee are proposed [19,20]. The recent efforts to restore the Dark bee from traces in the Czech sub-population in South Bohemia were tendentiously suppressed [21].

Beekeepers’ practices, such as the intentional introduction of different honey bee races, have increased genetic diversity in *Apis mellifera* populations all over Europe [22]. European beekeepers have historically introduced various honey bee races, leading to hybridization and the creation of new genetic clades [23]. The Carniolan honey bee is a subspecies of honey bee native to Europe that has been introduced to various regions worldwide due to its favorable features for beekeeping [15].

Microsatellites remain an affordable genetic marker with the capacity to capture multilocus genotype information, which can then be employed to estimate genetic diversity and population structure [24,25]. However, new genotyping and next-generation sequencing techniques have recently become available.

Nevertheless, there have been notable applications of microsatellite markers for the assessment of genetic variation, population structure, conservation genetics, and breeding of a wide range of organisms, including honeybees [26,27,28,29,30,31].

A recent study [32] based on haplotyping of the tRNA^leu^-cox2 region has demonstrated that the Czech Republic is exclusively inhabited by the C lineage of the honey bee. The occasional occurrence of individuals belonging to the A lineage has also been documented.

The genetic diversity and population structure of C lineages of honey bees (*A. m. ligustica*, *A. m. carnica*, *A. m. macedonica,* and others) have been evaluated in Slovenia, Croatia, Serbia, and Greece [26,29,33,34,35,36].

To address the current lack of knowledge regarding the molecular diversity of honeybee populations in the Czech Republic, we utilized a panel of 22 microsatellite loci to analyze the genetic variability and structure of honeybee populations across all locations where colonies are present. Samples were collected from both hive populations and floral sources, encompassing the entirety of the Czech Republic’s 77 districts.

## 2. Materials and Methods

### 2.1. Sampling of Bees

The aim was to cover the entire Czech Republic and its various geographical areas as evenly as possible during 2022–2023. The district was chosen as the basic territorial unit. There are 76 of these districts plus Prague (a list of districts with abbreviations and numerical code is in Table S1); they are administrative units that are relatively evenly distributed over the entire territory; the average size is 1024 km^2^.

**Figure 1:**
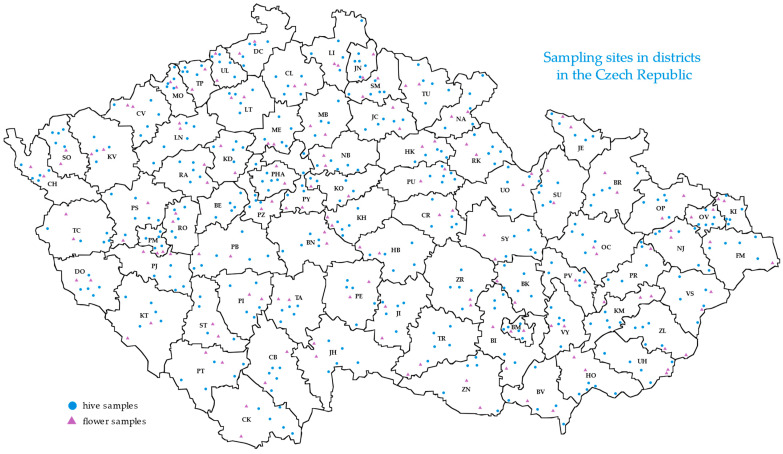
The map of sampling sites in individual districts in the Czech Republic. Abbreviations of districts: BE Beroun; BI Brno-venkov; BK Blansko; BM Brno-město; BN Benešov; BR Bruntál; BV Břeclav; CB České Budějovice; CK Český Krumlov; CL Česká Lípa; CR Chrudim; CV Chomutov; DC Děčín; DO Domažlice; FM Frýdek Místek; HB Havlíčkův Brod; HK Hradec Králové; HO Hodonín; CH Cheb; JC Jičín; JE Jeseník; JH Jindřichův Hradec; JI Jihlava; JN Jablonec nad Nisou; KI Karviná; KH Kutná Hora; KD Kladno; KM Kroměříž; KO Kolín; KT Klatovy; KV Karlovy Vary; LI Liberec; LN Louny; LT Litoměřice; MB Mladá Boleslav; ME Mělník; MO Most; NA Náchod; NB Nymburk; NJ Nový Jičín; OC Olomouc; OP Opava; OV Ostrava-město; PU Pardubice; PB Příbram; PE Pelhřimov; PY Praha-východ; PHA Praha; PI Písek; PJ Plzeň-jih; PM Plzeň-město; PR Přerov; PS Plzeň-sever; PT Prachatice; PV Prostějov; PZ Praha-západ; RA Rakovník; RK Rychnov nad Kněžnou; RO Rokycany; SM Semily; SO Sokolov; ST Strakonice; SU Šumperk; SY Svitavy; TA Tábor; TC Tachov; TP Teplice; TR Třebíč; TU Trutnov; UH Uherské Hradiště; UL Ústí nad Labem; UO Ústí nad Orlicí; VS Vsetín; VY Vyškov; ZL Zlín; ZN Znojmo; ZR Žďár nad Sázavou.

Collections from hives took place according to the scheme of five beekeepers per district, three colonies from each beekeeper, and 3–5 bees from one colony. The total number of bees analyzed from so-called “hive samples” reached 3647. In addition, the free-flying workers (unknown beekeeper and origin of workers) were also sampled by capture on flowers in the wild from places outside of settlements, at a distance of at least 2 km from the nearest hive, in the number of two locations per district and 3–5 workers per location, totally 553 bees were analyzed from so-called “flower samples”. The sampling sites are shown on the map of the Czech Republic in Figure 1.

After the sampling, the material was stored in the Genetic bank of Czech honeybees at a temperature of −20 °C until the DNA isolation.

### 2.2. DNA Extraction

Genomic DNA was extracted using a standard protocol (Tissue Genomic DNA Mini Kit, Geneaid, Taipei, Taiwan). Genomic DNA was extracted from thoracic muscle tissues.

### 2.3. PCR Amplification

Polymerase chain reaction (PCR) was performed using a thermocycler ABI Verity 96 Well (Applied Biosystems Inc., Foster City, CA, USA) in a total volume of 10 μL containing 1× Combi PPP Master Mix (Top-Bio, Vestec, Czech Republic), which contained 0.5 U of hot star Taq polymerase, 200 μM total dNTP and 2.5 mM MgCl_2_, and a specific amount of each primer and 0.5 μL of DNA isolate.

The microsatellite panel includes 22 microsatellite loci amplified in a total of four multiplexes. In Table S2a–d, the composition of individual multiplexes, the sequence of primers for a given microsatellite, their size, range, fluorescent label, repeating motif, and the first author are shown.

The concentration of individual primers in the multiplex reaction was optimized, and it was verified that the selected microsatellites were compatible with each other for the multiplex. The Multiplex 1 (6-plex) contained 0.1 μM Ap218, 0.1 μM A113, 0.1 μM A(B)024, 0.1 μM Ap249, 0.15 μM A088 and 0.2 μM Ap043 primers. The Multiplex 2 (8-plex) contained 0.1 μM A079, 0.1 μM Ac306, 0.1 μM Ap226, 0.1 μM A007, 0.1 μM Ap223, 0.2 μM Ap068, 0.2 μM A014 and 0.3 μM HB-C16-01 primers. The Multiplex 3 (5-plex) contained 0.1 μM AP019, 0.1 μM A(B)124, 0.1 μM Ap273, 0.2 μM Ap289 and 0.2 μM HB-C16-05 primers. The Multiplex 4 (3-plex) contained 0.2 μM A043, 0.1 μM Ap288 and 0.1 μM Ap049 primers.

The cycling conditions were as follows: 95 °C (2 min); 30 cycles of a 20 s denaturation at 95 °C, a 20 s annealing at 57 °C, a 30 s elongation at 72 °C; and a final extension step at 72 °C for 60 min.

### 2.4. Fragment Analysis

The four multiplex PCR products that were obtained were verified using agarose gel electrophoresis. Multiplexes 1 and 2 were analyzed separately, and multiplexes 3 and 4 were pooled. Fragment analysis was performed using ABI PRISM 3500 Genetic Analyser (Applied Biosystems) based on standard conditions (POP-7 polymer, G5 matrix). Fragment size was accurately determined with GeneScan™ 600 LIZ™ Size Standard and evaluated using GeneMapper v 6.0 software (Applied Biosystems).

### 2.5. Data Analysis

The calculations were performed in the GenAlEx version 6.5 environment [37]. The following parameters were calculated: the number of alleles (*Na*), the effective number of alleles (*Ne*), the Shannon information index (*I*), the observed (*Ho*), expected (*He*), and unbiased expected heterozygosity (*uHe*), and Fixation index (*F*). Wright’s F statistics (*F_ST_*, *F_IS,_* and *F_IT_*), as proposed by Weir and Cockerham [38], and the distribution of genetic diversity were analyzed using analysis of molecular variance (AMOVA). A paired t-test was performed in the R statistical computing environment, version 4.4.1 [39], to ascertain the statistical significance of the differences between the parameter values obtained from the hive and flower samples.

Pairwise Nei’s unbiased and pairwise *F_ST_* genetic distances between populations were calculated and used for principal component analysis (PCA) in GenAlEx version 6.5.

The Bayesian clustering method of STRUCTURE ver. 2.3.4 [40] was used to analyze the genetic diversity and degree of admixture of honeybee populations. Ten independent simulations were run, each including 10,000 burn-in steps followed by 100,000 Markov chain Monte Carlo (MCMC) iterations. Subsequently, we employed Clumpak ver. 1.1 [41] and Structure Selector [42], which implement Evanno method [43] and Puechmaille method [44] to ascertain the optimal number of clusters (K) that best fit the data, evaluating ΔK, MedMeaK, MaxMeaK, MedMedK, and MaxMedK. To determine the genetic structure and infer genetic admixture, discriminant analysis of principal components (DAPC) was conducted using the adegenet R package, version 2.1.10 [45]. This was performed within the R, version 4.4.1 [38].

## 3. Results

### 3.1. Genetic Diversity

The diversity results of the reference populations, based on the collection type from hives and flowers, are summarized in Table 1 and Table 2. All loci were polymorphic in both populations. The mean observed heterozygosity (*Ho*) for both populations was comparable to the expected heterozygosity (*He*). The average values for other parameters, such as effective population size (*Ne*) and Shannon’s information index (*I*), were also similar for both populations. However, the average fixation index (*F*) was higher in the hive sample (0.0483) compared to the flower sample (0.036).

The number of alleles detected in the studied hive sample ranged from four (locus AP274) to 20 alleles and above (loci A007, A014, HB-C16-01, and AP289). While in the flower sample, it ranged from four alleles (loci AP274 and AS(B)024) to 20 alleles and above (loci A014, HB-C16-01, and AP289).

The greatest genetic diversity, as indicated by the Shannon’s information index (*I*), was observed in both samples at locus HB-C16-01 (2.576 and 2.582), while the lowest value was found in both samples at locus Ap288 (0.418 and 0.382).

The lowest *Ho* and *He* (0.194–0.370 and 0.201–0.421, respectively) in the hive population were observed at loci Ap288, Ap273, and Ap049, and similarly low values were found at the same loci in the flower sample. The largest difference between *Ho* and *He* was at locus HB-C16-01 in both types of samples. The most heterozygous loci (*Ho* greater than 0.7) in the hive population were AP043, A007, A079, Ap068, and A(B)124, and in the flower sample were A007, A079, Ap068, HB-C16-01, and A(B)124. Fixation index values (*F*) higher than 0.1, indicating the difference between *Ho* and *He* and its relation to inbreeding, were observed in the hive sample at loci Ap218, HB-C16-01, A043, and Ap049, and in the flower sample at Ap218, HB-C16-01, and Ap049. The average diversity parameters of both sample groups are very similar and statistically non-significant (*p* > 0.05).

The majority of loci in the hive sample exhibited deviations from Hardy–Weinberg equilibrium, with only loci A(B)024 and Ac306 conforming to the equilibrium. Conversely, in the bees collected from flowers, most loci were not in Hardy–Weinberg equilibrium (only loci Ap113, Ap218, Ap226, HB-C16-01, A043, and Ap049 were found to be in equilibrium). Detailed results of the Hardy–Weinberg equilibrium analysis are presented in Table S3.

Table S4a,b provides a detailed description of the diversity parameters for all 77 districts sampled from hives and flowers. In samples from hives, the values of *Ho*, *He*, *I*, and *F* ranged from 0.493 (TA) to 0.587 (KT), from 0.530 (SM) to 0.608 (MB), from 1.104 (DO) to 1.279 (MB), and from −0.37 (UH) to 0.08 (RO), respectively. In samples from flowers, the values of *Ho*, *He*, *I*, and *F* ranged from 0.464 (PU) to 0.700 (KH), from 0.466 (HK) to 0.600 (ST), from 0.856 (JI) to 1.174 (VS), and from −0.266 (KH) to 0.122 (ZR), respectively. The results demonstrate that the high discrepancies between districts are not considerable. The only distinction between the populations based on the type of sample was observed in the F index. It was found that most of the samples from hives exhibited predominantly positive F index values, while the samples from flowers demonstrated a predominantly negative value.

*F*-statistics [38] for samples from hives and flowers are presented in Table 3. The *F_IS_* index, which measures the reduction in heterozygosity of an individual due to non-random mating in the subpopulation, was approximately zero for each locus in the sample from hives. Loci Ap289, Ap2018, A043, Ap049, and HB-C16-01 (with the highest value of 0.260) had *F_IS_* values above the average of *F_IS_* = 0.026. In the sample from flowers, *F_IS_* values were mainly negative, ranging from −0.124 (Ap223) to 0.082 (HB-C16-01). Conversely, A(B)024, HB-C16-05, A043, Ap049, Ap218, and HB-C16-01 had *F_IS_* values above the average of *F_IS_* = −0.054.

The *F_IT_* index values, indicating a reduction in individual heterozygosity due to non-random mating and population subdivision relative to the total population, ranged from 0.007 (Ac306) to 0.280 (HB-C16-01). Loci Ap218, A043, Ap049, and HB-C16-01 had *F_IT_* values above the average of *F_IT_* = 0.05. In the flower sample, *F_IT_* values ranged from −0.031 to 0.186, with loci A(B)024, HB-C16-05, A043, Ap049, Ap218, and HB-C16-01 having values above the average value *F_IT_* = 0.036.

The *F_ST_* index, representing the reduction of heterozygosity in a subpopulation due to random genetic drift, ranged from 0.019 (Ac306 and Ap289) to 0.032 (Ap049). Nine loci (A043, AP043, Ap226, HB-C16-01, Ap288, Ap249, A(B)024, Ap218, and Ap049) had *F_ST_* values above the average of *F_ST_* = 0.024. In the flower sample, *F_ST_* values ranged from 0.06 (Ap273) to 0.121 (Ap049), with six loci (A088, Ap226, A043, Ap218, HB-C16-01, Ap049) having *F_ST_* values above the average of *F_ST_* = 0.086.

Supplement Table S5a,b provides a detailed account of the frequencies of private alleles in individual populations (districts) collected from hives and flowers, respectively. There were more private alleles in the flower sample, which may be due to the smaller number of samples. In the populations from hives, locus A079 had the most private alleles (92, 118, 116, and 120) in different districts (BR, FM, JI, and PT), and in the samples from flowers, locus A(B)124 had the most private alleles (4, of which two alleles 210 and 242 were in the TP district).

### 3.2. Population Differentiation and Structure

The results of the AMOVA analysis indicated that most of the genetic variation was observed among individuals within population districts (samples from hives and from flowers, 91% and 93%, respectively). Conversely, only 2% and 1% of the variance, respectively, was attributed to among-population differences across the 77 districts (Table 4).

Results of principal component analysis (Table 5) of 77 populations sampled from hives and flowers indicate that the first three axes explained 36.59% and 29.23% of the variation (based on Nei’s pairwise distances) and 100% and 29% of the variation (based on *F_ST_* pairwise distances), respectively. The first axis explained 16.55% and 12.59% of the variation in samples from hives and flowers, respectively, based on Nei pairwise distances and 59.2% and 12.44% based on *F_ST_* pairwise distances.

**Figure 2:**
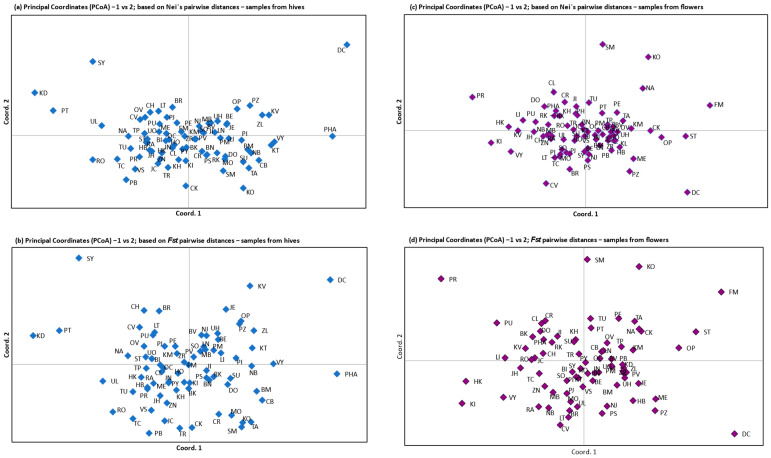
Principal component analysis (PCA) of 77 populations from hives (**a**,**b**) and from flower samples (**c**,**d**) based on Nei’s pairwise distances and *F_ST_* pairwise distances. The population pairwise Nei’s and *F_ST_* values for the samples from hives and flowers are presented in Tables S6 and S7, respectively.

The principal component analysis (Figure 2a–d), based on Nei’s pairwise distances and *F_ST_* pairwise distances in both types of samples (matrices are in Tables S6 and S7), indicated the absence of a significant structure, with all populations exhibiting overlapping patterns of data points. The PCA demonstrated that the genetic distance between the districts was relatively limited, with only a few populations exhibiting a moderate degree of genetic differentiation from the others (DC, PHA, SY, KD, and PT) from the hive collection. A slight degree of genetic differentiation from the flower sample was observed between districts (DC, PR, FM, SM, KO, and ST).

**Figure 3:**
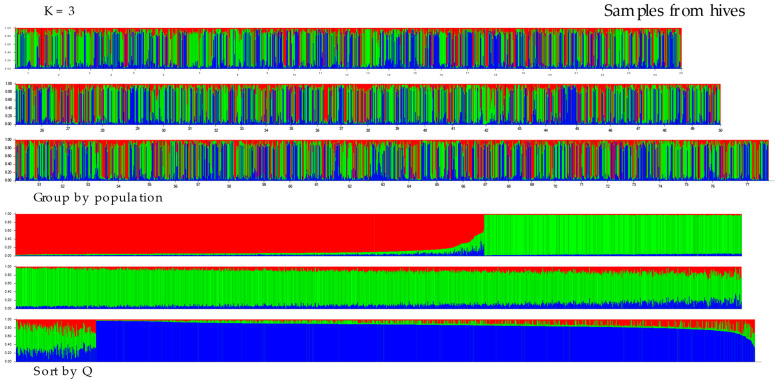
Proportions of inferred STRUCTURE clusters (optimal K = 3, based on Puechmaille and Evanno method in Figure S1a) from the individuals in samples from hives. Each vertical line denotes an individual sample, while the color indicates the probability of the individual belonging to a particular population district. The numbers (1–77) in the top chart represent districts (you can find the district designation in Table S1). In the Sort by Q graph, individuals are sorted according to their estimated membership in each population.

**Figure 4:**
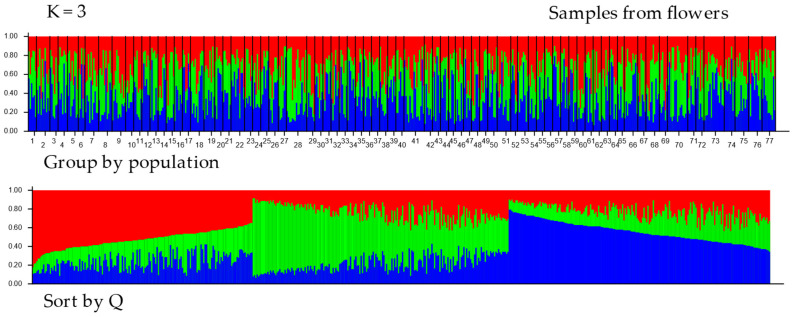
Proportions of inferred STRUCTURE clusters (optimal K = 3, based on the Puechmaille and Evanno method in Supplementary Figure S1b) from the individuals in samples from flowers. Each vertical line denotes an individual sample, while the color indicates the probability of the individual belonging to a particular population district. The numbers (1–77) in the top chart represent districts (you can find the district designation in Supplementary Table S1). In the Sort by Q graph, individuals are sorted according to their estimated membership in each population.

**Figure 5:**
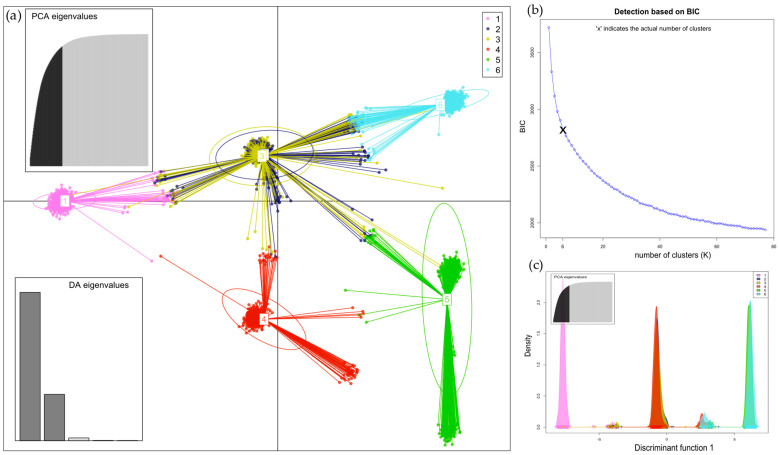
(**a**) Discriminant analysis of principal components (DAPC) of samples from hives found six clusters distributed across districts (Figure S2a). The plot describes the PCA eigenvalues, which explain how much variability is explained by the 1st and 2nd components, and the DA eigenvalues, which indicate how well each discriminant function separates clusters; (**b**) the plot shows the determination of the optimal number (K = 6) of clusters on the basis of the Bayes information criterion (BIC); and (**c**) the plot displays the densities of individuals on a discrimination function 1.

**Figure 6:**
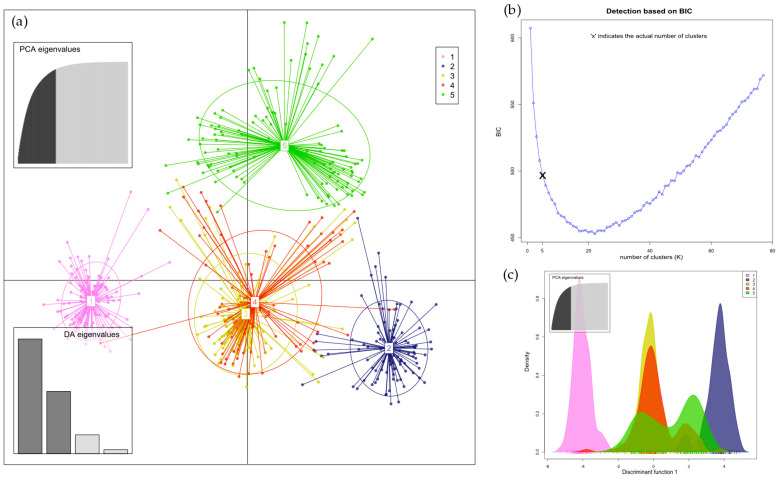
(**a**) Discriminant analysis of principal components (DAPC) of samples from flowers found five clusters distributed across districts (Figure S2b). The plot describes the PCA eigenvalues, which explain how much variability is explained by the 1st and 2nd components, and the DA eigenvalues, which indicate how well each discriminant function separates clusters; (**b**) the plot shows the determination of the optimal number (K = 5) of clusters on the basis of the Bayes information criterion (BIC); and (**c**) the plot displays the densities of individuals on a discrimination function 1.

The results of the structural analysis using STRUCTURE ver. 2.3.4 are shown in Figure 3 and Figure 4. The following analyses of the results from Structure using the Puechmaille (MedMeaK, MaxMeaK, MedMedK, MaxMedK) and Evanno methods (delta K), as implemented in the software Structure Selector and Clumpak ver. 1.1, respectively, indicated that the optimal value of K was 3 in both types of samples, from hives and from flowers (see Figure S1a,b). This finding suggests that the 77 geographical regions-districts should be divided into three genetically distinct subgroups based on microsatellite loci. All clusters derived by Structure are evenly distributed across all districts. Proportions of membership of each pre-defined population in each of the three clusters are presented in Table S8. Allele-frequency divergence was observed among three inferred clusters, ranging from 0.0226 to 0.0437 in samples from hives and from 0.0404 to 0.0774 in samples from flowers. Moreover, the results of the DAPC analysis indicated that there were six clusters in the hives, while five clusters were identified in the flower samples (see Figure 5 and Figure 6). The assignment of individuals from the districts to the different clusters is shown in Figure S2. Here, we can see that the original groups (districts) were not perfectly identified in the derived clusters.

## 4. Discussion

The genetic diversity of honeybees in the Czech Republic has not been comprehensively studied using nuclear highly polymorphic markers (microsatellites) across the entire geographic area of the country. This study represents the most thorough and detailed analysis to date conducted in the Central European region.

A total of 22 microsatellite loci were analyzed in 77 subpopulations (geographical regions-districts), revealing a high degree of diversity in the whole population. However, no distinctive differences between districts were found. This is likely due to the relatively small size of the districts and the high degree of breeding, which causes a high degree of uniformity in high-diversity breeds.

The natural mating process, which is often exploited by beekeepers, and polyandry are among the natural effects that will have an impact. In comparison to the source population of *A. m. carnica* (especially in Slovenia), the Czech Republic has a limited number of queen bees that have been introduced into the country’s honeybee population. This replacement of the original black bee population has been occurring gradually over the past two centuries [12,13], with imports of queen bees taking place across this period [11].

Similar values of heterozygosity (approximately 0.55) to those observed in our study were reported by [29,46] for *A. m. carnica* in Central and Southeastern Europe. Lower heterozygosity values (0.45) have been documented for *A. m. carnica* in Serbia [34]. Conversely, higher heterozygosity values (above 0.6) have been observed for *A. m. carnica* in Hungary, Poland, and Slovakia [29,47,48], as well as in Croatia [26] and in Kazakhstan where the presence of even five honeybee subspecies is supposed [49].

The mean number of alleles identified in our sample set varies according to the method of sample collection, with a mean of 13.77 in hives and 11.18 in flowers. The smaller number of alleles collected from flowers can be explained by the overall lower number of samples collected from flowers compared to samples collected from hives. However, in the case of a comparable number, we would expect a higher diversity in samples collected from flowers. The diversity parameter Na in our population is similar to that of *Apis mellifera carnica* described in Central and Southeastern Europe (13.82) [46]. In the Serbian population (which belongs to the C lineage native area with *A. m. carnic*a and *A. m. macedonica*), however, the Na value was lower, in the range of 5.8–9.0, depending on the locality [29].

In other subspecies of honeybees, the degree of diversity varies. For example, an analysis of 19 microsatellites of the subspecies *A. m. scutellata* and *A. m. capensis* in South Africa revealed a high level of variability based on an average number of alleles per locus (10.23 and 9.94, respectively) and on observed heterozygosity values (0.76 and 0.75, respectively) [28]. They showed two distinct evolutionary units in the Republic of South Africa, though the results did not match those of earlier morphometric and molecular analyses, suggesting that the microsatellites they tested were not sufficient for subspecies identification purposes. High diversity was also described from Turkey, with an average number of alleles of 16.1 and an average observed heterozygosity of 0.54 [50]. In contrast, the diversity (heterozygosity 0.15–0.22) in the *A. m. meda* in Iran was found to be markedly low [51]. The mean number of alleles per locus in our population was 13.77 and 11.18, while in Iran, it ranged from 4.0 to 7.9. The observed heterozygosities ranged from 0.13 to 0.22 across the various Iranian regions. Yildiz et al. [52] investigated the genetic diversity of *A. m. caucasia* in Turkey, utilizing 30 microsatellite loci (equivalent to our 22 loci). They observed an extremely low level of heterozygosity (0.03), which they attributed to the prevalence of inbreeding.

Nevertheless, no correlation has been identified between the genetic clusters and the corresponding geographical areas (districts) in which they are distributed. The principal component analysis demonstrated that the genetic distance between the districts was relatively limited, with only a few districts exhibiting a notable divergence from the other populations that do not correspond with geographical distribution or historic importation of non-native queens [12]. Molecular diversity is relatively evenly distributed across all districts of the country, probably due to the high density of honeybee colonies [1,3,4,5]. Even in the border areas, where some gene flow from neighboring countries might be expected, molecular diversity was similar to those placed inland. However, some degree of genetic differentiation and clustering was confirmed. This is probably a consequence of the gradual random admixture of breeding material from different countries (Slovenia, Croatia, Austria, Germany, etc.) and its subsequent spread throughout the Czech Republic. Based on historical data [11,12,16], we assume that several different genetic lines have been used in succession, whose offspring then form the above-mentioned clusters of individuals occurring in all districts. Natural mating and polyandry with high colony densities also have a supporting effect. Similarly, the low differentiation observed in *A. m. carnica* is also explained in Slovenia [33] and in *A. m. meda* in Iran [51].

## 5. Conclusions

Our findings indicate that honeybee populations in the Czech Republic exhibit a high level of genetic variability attributable to a highly mixed genetic pool. This is related to the history of bee breeding and rearing in this region over the last 200 years. While minor local patterns of genetic diversity were identified, no strict boundaries between geographical (districts) and genetic groups (clusters) were observed. The analysis confirmed a certain genetic differentiation and clustering. This is believed to be the result of multiple uncontrolled interbreeding and numerous instances of maternal imports and their subsequent spread throughout the Czech Republic. However, further investigation into additional sources and mechanisms of admixture is necessary.

## Figures and Tables

**Table 1:** Parameters of the genetic diversity of *Apis mellifera* collected from hives.

Locus	*N*	*Na*	*Ne*	*I*	*Ho*	*He*	*uHe*	*F*	*HWE*
A(B)024	3626	5	2.640	1.026	0.608	0.621	0.621	0.022	ns
A088	3637	14	2.288	1.194	0.547	0.563	0.563	0.029	**
Ap043	3568	14	4.072	1.634	0.718	0.754	0.755	0.049	***
Ap113	3643	18	1.767	0.951	0.429	0.434	0.434	0.011	***
Ap218	3412	5	2.680	1.042	0.562	0.627	0.627	0.103	***
Ap249	3635	12	1.868	0.865	0.451	0.465	0.465	0.030	***
A007	3636	25	4.511	1.838	0.762	0.778	0.778	0.022	***
A014	3636	26	2.030	1.290	0.502	0.508	0.508	0.011	***
A079	3517	17	5.986	1.968	0.819	0.833	0.833	0.017	***
Ac306	3640	13	2.007	1.104	0.498	0.502	0.502	0.008	ns
Ap068	3632	10	3.597	1.518	0.703	0.722	0.722	0.026	***
Ap223	3544	7	1.722	0.796	0.413	0.419	0.419	0.016	***
Ap226	3606	10	2.975	1.249	0.655	0.664	0.664	0.013	***
HB-C16-01	3292	24	10.501	2.576	0.649	0.905	0.905	0.283	***
A(B)124	3553	17	4.007	1.809	0.740	0.750	0.751	0.014	***
AP019	3570	10	2.718	1.271	0.608	0.632	0.632	0.038	***
Ap273	3643	4	1.487	0.645	0.318	0.328	0.328	0.031	*
Ap289	3637	27	1.981	1.344	0.472	0.495	0.495	0.047	***
HB-C16-05	3615	19	2.236	1.389	0.538	0.553	0.553	0.027	***
A043	3513	12	2.363	1.223	0.513	0.577	0.577	0.111	***
Ap049	3551	8	1.728	0.905	0.370	0.421	0.421	0.121	***
Ap288	3544	6	1.252	0.418	0.194	0.201	0.201	0.037	***
Mean	3575	13.7727	3.0189	1.2752	0.5485	0.5796	0.5797	0.0483	
SE	18.466	1.5146	0.4318	0.1031	0.0329	0.0365	0.0365	0.0131	

*N*, number of samples; *Na*, No. of different alleles; *Ne*, No. of effective alleles = 1/(Sum p_i_^2^); *I*, Shannon’s information index = −1 × Sum (p_i_ × Ln (p_i_)); *Ho*, observed heterozygosity = No. of Hets/N; *He*, expected heterozygosity = 1 − Sum p_i_^2^; *uHe*, unbiased expected heterozygosity = (2N/(2N − 1)) × *He*; *F*, fixation index = (*He* − *Ho*)/*He* = 1 − (*Ho*/*He*); where p_i_ is the frequency of the ith allele for the population; *HWE*, Chi-square tests for Hardy–Weinberg equilibrium, ns = not significant, * *p* < 0.05, ** *p* < 0.01, *** *p* < 0.001 (see Table S3a).

**Table 2:** Parameters of the genetic diversity of *Apis mellifera* collected from flowers.

Locus	*N*	*Na*	*Ne*	*I*	*Ho*	*He*	*uHe*	*F*	*HWE*
A(B)024	545	4	2.605	1.018	0.591	0.616	0.617	0.041	ns
A088	546	10	2.296	1.174	0.551	0.564	0.565	0.023	ns
Ap043	543	10	3.984	1.593	0.729	0.749	0.750	0.026	ns
Ap113	550	16	1.770	0.934	0.438	0.435	0.435	−0.007	***
Ap218	542	5	2.700	1.060	0.546	0.630	0.630	0.133	***
Ap249	550	8	1.847	0.832	0.467	0.459	0.459	−0.019	ns
A007	548	15	4.374	1.765	0.765	0.771	0.772	0.009	ns
A014	548	22	2.023	1.298	0.500	0.506	0.506	0.011	ns
A079	548	13	6.169	1.992	0.816	0.838	0.839	0.026	ns
Ac306	548	11	1.975	1.076	0.484	0.494	0.494	0.020	ns
Ap068	547	11	3.589	1.514	0.702	0.721	0.722	0.027	ns
Ap223	549	5	1.744	0.796	0.439	0.427	0.427	−0.029	ns
Ap226	549	9	3.093	1.310	0.676	0.677	0.677	0.001	***
HB-C16-01	511	20	10.989	2.582	0.738	0.909	0.910	0.188	***
A(B)124	532	17	4.283	1.844	0.774	0.767	0.767	−0.010	ns
AP019	541	7	2.815	1.290	0.647	0.645	0.645	−0.003	ns
Ap273	551	4	1.466	0.624	0.318	0.318	0.318	0.001	ns
Ap289	552	24	1.942	1.317	0.476	0.485	0.485	0.018	ns
HB-C16-05	546	17	2.305	1.379	0.535	0.566	0.567	0.055	ns
A043	541	8	2.250	1.162	0.505	0.556	0.556	0.092	***
Ap049	541	6	1.767	0.926	0.370	0.434	0.434	0.148	***
Ap288	541	4	1.218	0.382	0.174	0.179	0.179	0.031	ns
Mean	544.045	11.182	3.055	1.267	0.556	0.579	0.580	0.036	
SE	1.856	1.292	0.454	0.104	0.035	0.037	0.037	0.012	

*N*, number of samples; *Na*, No. of different alleles; *Ne*, No. of effective alleles = 1/(Sum p_i_^2^); *I*, Shannon’s information index = −1 × Sum (p_i_ × Ln (p_i_)); *Ho*, observed heterozygosity = No. of Hets/N; *He*, expected heterozygosity = 1 − Sum p_i_^2^; *uHe*, unbiased expected heterozygosity = (2N/(2N − 1)) × *He*; *F*, fixation index = (*He* − *Ho*)/*He* = 1 − (*Ho*/*He*); where p_i_ is the frequency of the ith allele for the population; *WHE*, Chi-square tests for Hardy–Weinberg equilibrium, ns = not significant, *** *p* < 0.001 (see Table S3b).

**Table 3:** F Summary of *F*-statistics for all loci over the 77 subpopulations (districts).

	Hive Samples			Flower Samples		
Locus	*F_IS_*	*F_IT_*	*F_ST_*	*F_IS_*	*F_IT_*	*F_ST_*
A(B)024	−0.001	0.026	0.027	−0.041	0.047	0.085
A088	0.010	0.031	0.021	−0.096	0.000	0.087
AP043	0.022	0.046	0.025	−0.067	0.021	0.083
Ap113	−0.011	0.014	0.024	−0.092	−0.005	0.080
Ap218	0.080	0.108	0.030	0.059	0.150	0.097
Ap249	0.005	0.031	0.026	−0.100	−0.011	0.081
A007	−0.001	0.023	0.023	−0.067	0.017	0.079
A014	−0.013	0.010	0.023	−0.085	0.004	0.082
A079	−0.007	0.017	0.024	−0.061	0.030	0.086
Ac306	−0.012	0.007	0.019	−0.060	0.024	0.079
Ap068	0.002	0.025	0.024	−0.063	0.028	0.086
Ap223	−0.006	0.015	0.021	−0.124	−0.031	0.082
Ap226	−0.015	0.011	0.025	−0.110	−0.008	0.092
HB-C16-01	0.260	0.280	0.026	0.082	0.186	0.113
A(B)124	−0.005	0.018	0.023	−0.108	−0.018	0.080
AP019	0.013	0.037	0.024	−0.084	0.005	0.083
Ap273	0.008	0.030	0.022	−0.072	−0.007	0.060
Ap289	0.031	0.049	0.019	−0.064	0.019	0.078
HB-C16-05	0.010	0.030	0.020	−0.030	0.058	0.085
A043	0.093	0.116	0.025	0.018	0.110	0.093
Ap049	0.104	0.132	0.032	0.039	0.155	0.121
Ap288	0.009	0.036	0.026	−0.066	0.015	0.076
Mean	0.026	0.050	0.024	−0.054	0.036	0.086
SE	0.013	0.013	0.001	0.012	0.013	0.003

*F_IS_* = (Mean *He* − Mean *Ho*)/Mean *He*; *F_IT_* = (*Ht* − Mean *Ho*)/*Ht*; *F_ST_* = (*Ht* − Mean *He*)/*Ht*; where Mean *He* = Average *He* across the populations; Mean *Ho* = Average *Ho* across the populations; *Ht* = Total Expected Heterozygosity = 1 − ∑tp_i_^2^ where tp_i_ is the frequency of the ith allele for the total and Sum tp_i_^2^ is the sum of the squared total allele frequencies.

**Table 4:** Analysis of molecular variance of the genetic partitioning of honeybees for 77 populations (districts) in the Czech Republic.

Source	df	Sum of Squares	Mean of Squares	Estimated Variance	Percentage of Variation (%)
AMOVA (Hive Sample)					
Between regional populations	77	1251.921	16.259	0.100	2
Among Individuals	3569	24,695.233	6.919	0.505	8
Within Individuals	3647	21,548.000	5.908	5.908	91
Total	7293	47,495.155		6.514	100
AMOVA (Flower sample)					
Between regional populations	77	619.940	8.157	0.095	1
Among Individuals	476	3231.345	6.789	0.388	6
Within Individuals	553	3325.000	6.013	6.013	93
Total	1105	7176.285		6.496	100

Sources of variation are described as follows: ‘between populations’ refers to genetic variation partitioned across 77 districts; ‘among individuals within populations’ refers to genetic variation partitioned among individuals within a population; and ‘within individuals’ refers to genetic variation partitioned within each individual.

**Table 5:** Percentage of variation explained by the first three axes from Principal Coordinates Analysis (PCoA).

	PCoA via Nei’s Pairwise Distance			PCoA via FST Pairwise Distance		
Axis	1	2	3	1	2	3
From hives						
%	16.55	10.82	9.22	59.20	40.80	0.00
Cum. %	16.55	27.37	36.59	59.20	100.00	100.00
From flowers						
%	12.59	9.15	7.49	12.44	8.61	7.94
Cum. %	12.59	21.74	29.23	12.44	21.06	29.00

## Data Availability

Data are available on request from the corresponding author.

## Supplementary Materials

The following supporting information can be downloaded at https://www.mdpi.com/article/10.3390/insects16010055/s1, Figure S1: The optimal K determined through the utilization of the Clumpak and Structure Selector (a). MedMeaK, MaxMeaK, MedMedK, and MaxMedK (Puechmaille method), (b) and delta K (Evanno method) for the assumed number of genetic clusters in samples from hives (Figure S1a) and from flowers (Figure S1b); Figure S2: DAPC analysis—Membership of individuals to the different clusters in samples from hives (Figure S2a) and from flowers (Figure S2b). Rows correspond to actual groups (districts), while columns correspond to inferred groups (“inf”). Table S1: List of districts, abbreviation and code number; Table S2: Microsatellite loci and multiplex (1–4) primer group; Table S3: Summary of Chi-Square Tests for Hardy–Weinberg Equilibrium in samples from hives (Table S3a) and from flowers (Table S3b); Table S4: Parameters of the genetic diversity of *Apis mellifera* in 77 districts collected from hives (Table S4a) and from flowers (Table S4b); Table S5: Summary of private alleles by sample collected from hives (Table S5a) and from flowers (Table S5b); Table S6: Nei’s pairwise distances and *F_ST_* pairwise distances in sample from hives; Table S7: Nei’s pairwise distances and *F_ST_* pairwise distances in sample from flowers; Table S8: Detailed results from Structure analysis: Proportion of membership of each pre-defined population in each of the three clusters; Allele-freq. divergence among pops (Net nucleotide distance), computed using point estimates of P through structure analysis; Average distances (expected heterozygosity) between individuals in the same cluster.
