# Supplementary material for: Assessing Genetic Diversity and Population Structure of Western Honey Bees in the Czech Republic Using 22 Microsatellite Loci
Source: Insects. 2025 Jan 9;16(1):55. doi: 10.3390/insects16010055 (PMC11766434; doi:10.3390/insects16010055)
Supplement: Supplementary file 1 [file insects-16-00055-s001.zip › Figure S1 a-b.pdf]

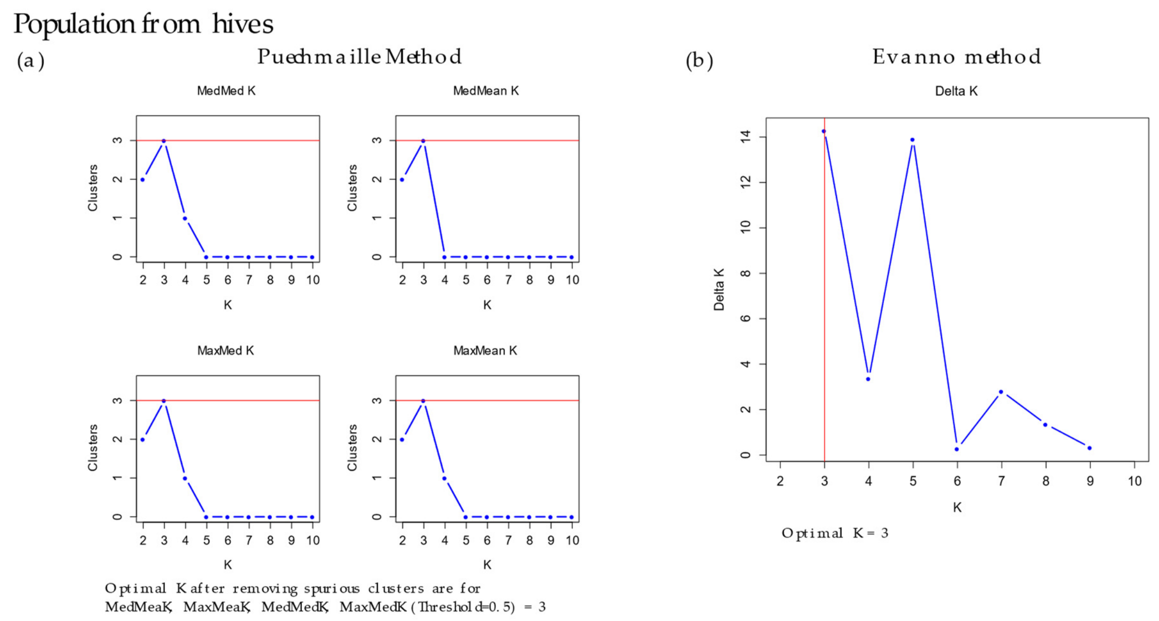

Figure S1a: The optimal K determined through the utilization of the Clumpak and Structure Selector (**a**). MedMeaK, MaxMeaK, MedMedK, and MaxMedK (Puechmaille method), (**b**) and delta K (Evanno method) for the assumed number of genetic clusters in population from hives.

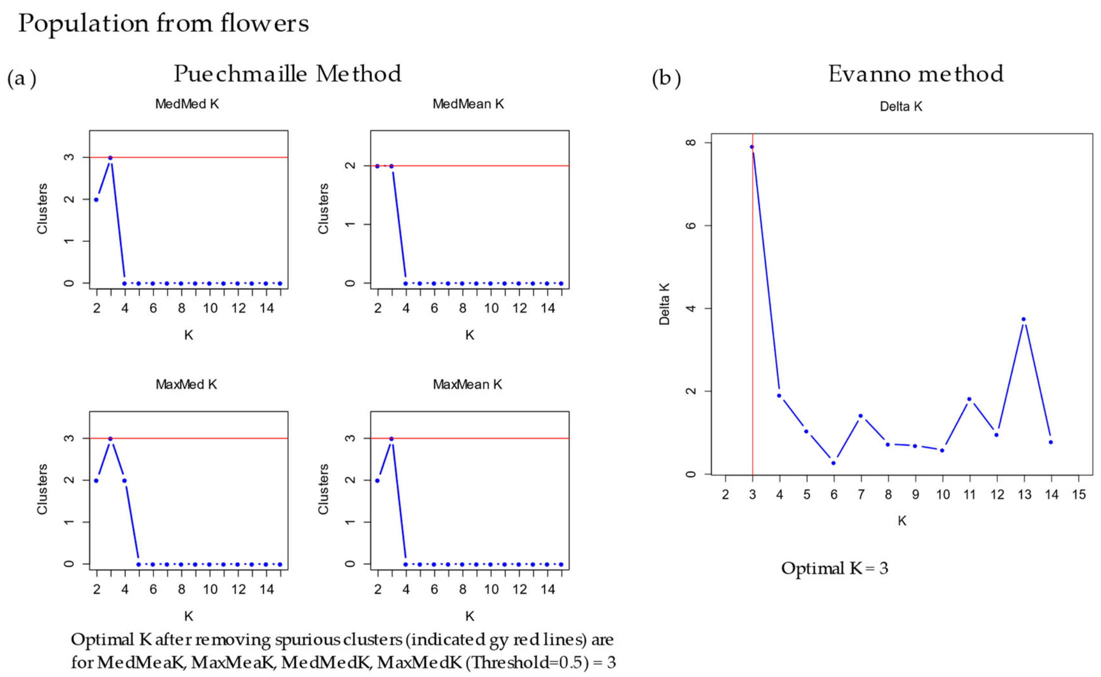

Figure S1b: The optimal K determined through the utilization of the Clumpak and Structure Selector (**a**) MedMeaK, MaxMeaK, MedMedK, and MaxMedK (Puechmaille method), (**b**) and delta K (Evanno method) for the assumed number of genetic clusters in population from flowers.
