# Supplementary material for: Assessing Genetic Diversity and Population Structure of Western Honey Bees in the Czech Republic Using 22 Microsatellite Loci
Source: Insects. 2025 Jan 9;16(1):55. doi: 10.3390/insects16010055 (PMC11766434; doi:10.3390/insects16010055)
Supplement: Supplementary file 1 [file insects-16-00055-s001.zip › Figure S2 a-b.pdf]

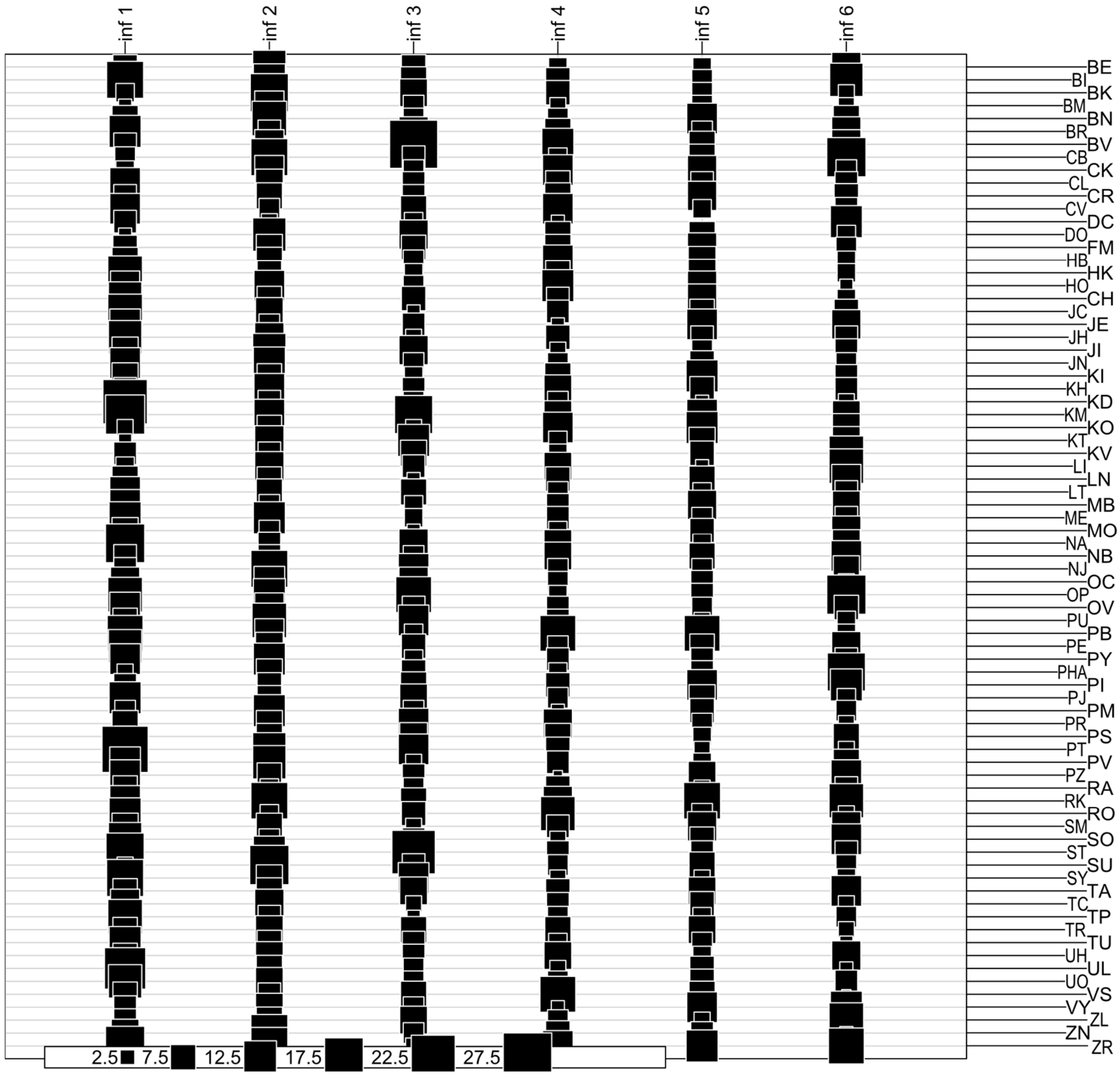

Figure S2a: DAPC analysis - Membership of individuals to the different clusters in populations from hives. Rows correspond to actual groups (districts), while columns correspond to inferred groups ("inf").

DAPC analysis – populations from hives (outputs)

```
> klastr$Kstat
     K=1      K=2      K=3      K=4      K=5      K=6      K=7      K=8      K=9     K=10     K=11     K=12
3721.020 3331.399 3117.425 2980.560 2904.278 2816.891 2766.409 2722.494 2682.720 2644.474 2605.997 2573.078
    K=13     K=14     K=15     K=16     K=17     K=18     K=19     K=20     K=21     K=22     K=23     K=24
2542.057 2517.935 2489.963 2458.573 2434.672 2411.025 2397.047 2371.005 2352.809 2337.617 2316.329 2298.713
    K=25     K=26     K=27     K=28     K=29     K=30     K=31     K=32     K=33     K=34     K=35     K=36
2291.068 2271.678 2253.366 2240.173 2232.176 2219.597 2205.768 2195.659 2172.475 2166.667 2158.383 2153.038
    K=37     K=38     K=39     K=40     K=41     K=42     K=43     K=44     K=45     K=46     K=47     K=48
2148.553 2128.224 2126.193 2111.029 2107.983 2096.546 2082.100 2077.470 2075.102 2070.789 2060.234 2054.364
    K=49     K=50     K=51     K=52     K=53     K=54     K=55     K=56     K=57     K=58     K=59     K=60
2055.142 2042.653 2033.552 2031.313 2027.558 2020.394 2024.639 2011.566 2011.697 2004.579 1996.652 1992.394
    K=61     K=62     K=63     K=64     K=65     K=66     K=67     K=68     K=69     K=70     K=71     K=72
1995.125 1989.844 1983.643 1979.997 1978.433 1974.641 1972.468 1971.741 1960.609 1957.601 1952.786 1949.607
    K=73     K=74     K=75     K=76     K=77
1950.520 1948.992 1948.951 1946.295 1938.534
> klastr$stat
     K=6
2816.891
> klastr$size
[1] 749 726 545 512 540 575
```

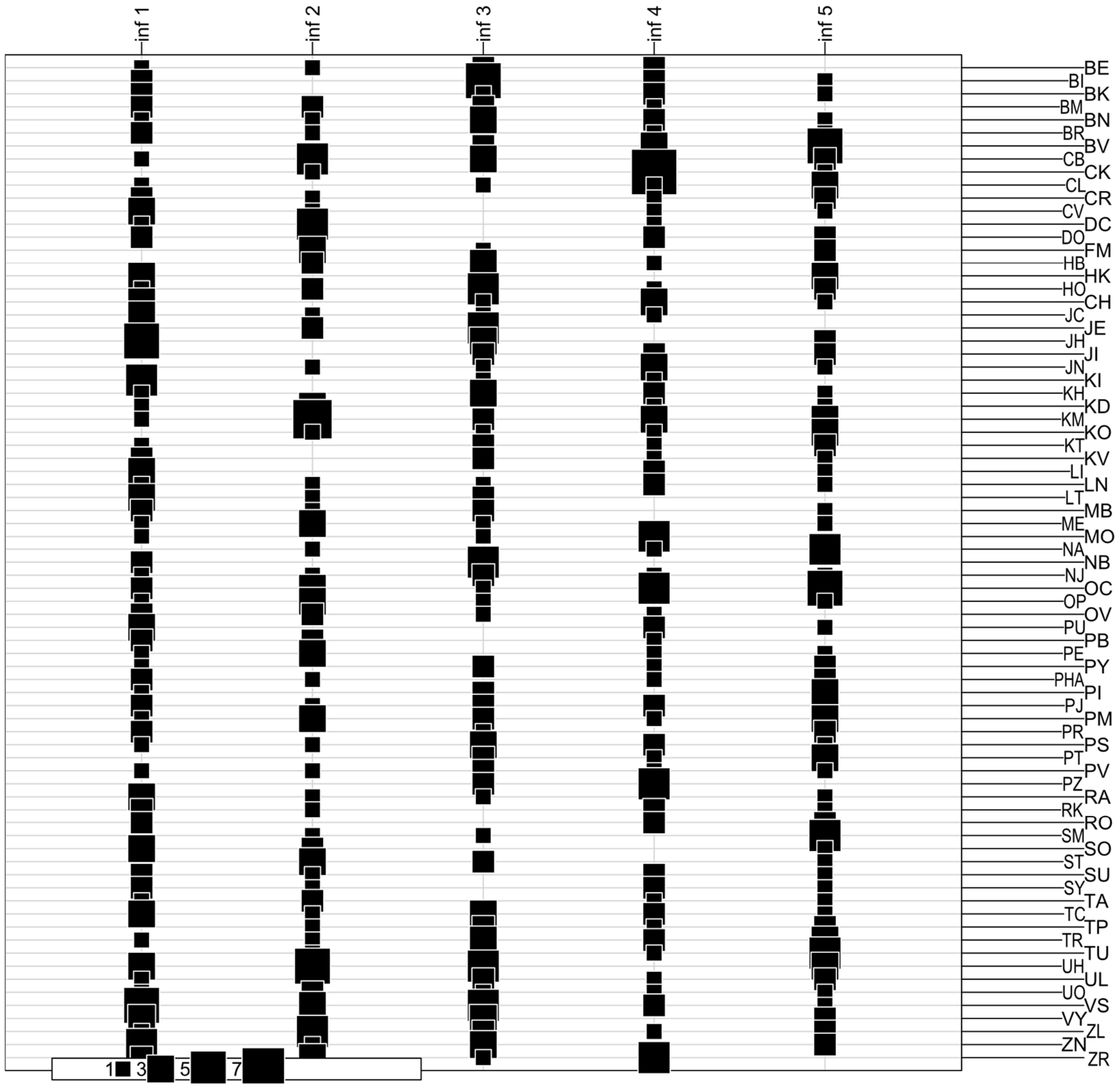

Figure S2b: DAPC analysis - Membership of individuals to the different clusters in populations from flowers. Rows correspond to actual groups (districts), while columns correspond to inferred groups ("inf").

DAPC analysis – populations from flowers (outputs)

```
> klastr$Kstat
     K=1      K=2      K=3      K=4      K=5      K=6      K=7      K=8      K=9     K=10     K=11     K=12 
607.3360 551.1725 525.8362 507.8990 496.6399 489.2211 483.5743 478.4925 475.3379 468.4581 466.3850 465.4080 
    K=13     K=14     K=15     K=16     K=17     K=18     K=19     K=20     K=21     K=22     K=23     K=24 
461.7272 460.6783 458.6212 457.8098 454.9169 455.0385 455.5805 454.0927 454.6082 452.8863 455.1719 455.3054 
    K=25     K=26     K=27     K=28     K=29     K=30     K=31     K=32     K=33     K=34     K=35     K=36 
455.0364 457.7641 458.2846 460.0051 461.4440 459.0999 462.3456 462.9773 464.4044 466.0390 468.8516 469.9138 
    K=37     K=38     K=39     K=40     K=41     K=42     K=43     K=44     K=45     K=46     K=47     K=48 
470.6252 474.1354 476.3680 475.3458 478.4196 479.9324 484.5318 482.3982 488.8047 489.1516 493.0745 492.5618 
    K=49     K=50     K=51     K=52     K=53     K=54     K=55     K=56     K=57     K=58     K=59     K=60 
498.8374 498.0805 500.0716 503.7559 504.3173 507.1361 511.6899 510.4048 514.4801 517.8972 520.7473 523.2697 
    K=61     K=62     K=63     K=64     K=65     K=66     K=67     K=68     K=69     K=70     K=71     K=72 
526.5333 529.6288 530.4333 532.9193 534.9219 539.6296 542.7008 544.6371 548.2316 551.9753 552.8153 555.0701 
    K=73     K=74     K=75     K=76     K=77 
558.6063 561.5738 561.7971 569.0629 571.9845 
> klastr$stat
     K=5 
496.6399 
> klastr$size
[1] 120  99 116 104 114
```
