# Supplementary material for: Assessing Genetic Diversity and Population Structure of Western Honey Bees in the Czech Republic Using 22 Microsatellite Loci
Source: Insects. 2025 Jan 9;16(1):55. doi: 10.3390/insects16010055 (PMC11766434; doi:10.3390/insects16010055)
Supplement: Supplementary file 1 [file insects-16-00055-s001.zip › Table S2 a-d.pdf]

Table S2a. Microsatellite loci and multiplex 1 primer group

Primer (locus)r	Sequence	Length (nt)	Range (bp)	Fluorescent label	Motif	Author
**Ap218-F**	AGGGATGGAATTCTTCGATT	20	122-138	6-FAM	(CA)6	Solignac et al., 2003
**Ap218-R**	TTGTCACAATTCCGCTTGA	19				
**A113-F**	CTCGAATCGTGGCGTCC	17	195-237	6-FAM	(TC)2C(TC)2TT(TC)5TT (TC)8TT(TC)5	Estoup et al. 1995
**A113-R**	CCTGTATTTTGCAACCTCGC	20				
**A024-F**	CACAAGTTCCAACAATGC	18	92-104	VIC	(CT)11	Estoup et al. 1995
**A024-R**	CACATTGAGGATGAGCG	17				
**Ap249-F**	CGCGCGACGACGAAATGT	18	207-220	VIC	(GA)6(GA)8	Solignac et al., 2003
**Ap249-R**	CAGTCCTTTGATTCGCGCTACC	22				
**A088-F**	CGAATTAACCGATTTGTCG	19	131-152	NED	(CT)10TC(CCTT)2(CTTT)3(GGA)7	Estoup et al. 1995
**A088-R**	GATCGCAATTATTGAAGGAG	20				
**Ap043-F**	GGCGTGCACAGCTTATTCC	19	134-162	PET	(TA)6GATA(GA)10	Garnery et al. 1998
**Ap043-R**	CGAAGGTGGTTTCAGGCC	18				

Table S2b. Microsatellite loci and multiplex 2 primer group

Primer (locus)r	Sequence	Length (nt)	Range (bp)	Fluorescent label	Motif	Author
**A079-F**	CGAAGGTTGCGGAGTCCTC	19	89-124	6-FAM	$(CT)_{14}$	Estoup et al. 1994
**A079-R**	GTCGTCGGACCGATGCG	17				
**Ac306-F**	GAATATGCCGCTGCCACC	18	161-187	6-FAM	$(CT)_{11}$	Solignac et al., 2003
**Ac306-R**	TTTCGTTGCATCCGAGCG	18				
**Ap226-F**	AACGGTGTTCGCGAAACG	18	228-248	6-FAM	$(CT)_{8}$	Solignac et al., 2003
**Ap226-R**	AGCCAACTCGTGCGGTCA	18				
**A007-F**	CCCTTCCTCTTTCATCTTCC	20	94-136	VIC	$(CT)_{3}(T)_{7}CTTCG(CT)_{24}$	Estoup et al. 1994
**A007-R**	GTTAGTGCCCTCCTCTTGC	19				
**HB-C16-01-F**	AAAATGCGATTCTAATCTGG	20	249-309	VIC	$(GA)_{35}$	Shaibi et al.2008
**HB-C16-01-R**	TTGCCTAAAATGCTTGCTAT	20				
**Ap068-F**	TGTCTGCCCTCCTCTCTGTT	20	147-169	NED	$(CT)_{12}(TA)_{8}$	Solignac et al., 2003
**Ap068-R**	CACATCGAGCGAGAAGGC	18				
**A014-F**	GTGTCGCAATCGACGTAACC	20	214-247	NED	$(CT)_{13}(GGT)_{9}$	Estoup et al. 1994
**A014-R**	GTCGATTACCGATCGTGACG	20				
**Ap223-F**	TCGTACAACGTCGCGCAA	18	147-182	PET	$(T)_{5}(C)_{4}A(T)6(C_{5})$	Solignac et al., 2003
**Ap223-R**	GCCGCTCGCCTGTATCTG	18				

Table S2c. Microsatellite loci and multiplex 3 primer group

Primer (locus)r	Sequence	Length (nt)	Rozsah (bp)	Fluorescent label	Motif	Author
**Ap019-F**	CTCGTTTCTTCCATTGCG	18	133-144	6-FAM	$(TC)_{11}$	Tunca 2009
**Ap019-R**	CGGTACGCGGTAGAAAGA	18				
**B124-F**	GCAACAGGTCGGGTTAGAG	19	210-244	6-FAM	$(CT)_8$ $(CT)_{14}$CCTC $(GC)_3(GGCT)_8$	Estoup et al. 1994
**B124-R**	CAGGATAGGGTAGGTAAGCAG	21				
**Ap273-F**	GATCTTGTGTTAAACAGCCG	20	102-108	NED	$(CT)_8$	Solignac et al., 2003
**Ap273-R**	GATCTCTGGCAGACGAAGAG	20				
**Ap289-F**	AGCTAGGTCTTTCTAAGAGTGTTG	24	173-217	NED	$(GA)_5$	Solignac et al., 2003
**Ap289-R**	TTCGACCGCAATAACATTC	19				
**HB-C16-05-F**	ATTTTATGVGVGTTTCGTA	19	70-110	PET	$(TC)_{23}$	Shaibi et al.2008
**HB-C16-05-R**	CATGGCTCCTCCATTAAATC	20				

Table S2d. Microsatellite loci and multiplex 4 primer group

Primer (locus)r	Sequence	Length (nt)	Rozsah (bp)	Fluorescent label	Motif	Author
**Ap049-F**	GGGCTTCGTACGTCCACC	18	121-151	6-FAM	$(AGG)_7$	Solignac et al., 2003
**Ap049-R**	GGGCTTCGTACGTCCACC	18				
**A043-F**	CCGCTCATTAAGATATCCG	19	123-143	VIC	$(CT)_{13}$	Estoup et al. 1994
**A043-R**	CCGCTCATTAAGATATCCG	19				
**Ap288-F**	GTTAGTTCGTCGTCGACCG	19	125-135	NED	$(TAA)_8$	Solignac et al., 2003
**Ap288-R**	TCTTAGCTTTATAACGAGCACG	22				
