# Supplementary material for: Assessing Genetic Diversity and Population Structure of Western Honey Bees in the Czech Republic Using 22 Microsatellite Loci
Source: Insects. 2025 Jan 9;16(1):55. doi: 10.3390/insects16010055 (PMC11766434; doi:10.3390/insects16010055)
Supplement: Supplementary file 1 [file insects-16-00055-s001.zip › Table S3 a-b.pdf]

Table S3a: Summary of Chi-Square Tests for Hardy-Weinberg Equilibrium in samples from hives

Locus	DF	ChiSq	Prob	Signif
**A(B)024**	10	8.582	0.572	ns
**A088**	91	128.445	0.006	**
**AP043**	91	625.979	0.000	***
**Ap113**	153	251.780	0.000	***
**Ap218**	10	927.349	0.000	***
**Ap249**	66	1088.023	0.000	***
**A007**	300	2272.647	0.000	***
**A014**	325	705.570	0.000	***
**A079**	136	542.621	0.000	***
**Ac306**	78	65.152	0.850	ns
**Ap068**	45	175.879	0.000	***
**Ap223**	21	412.997	0.000	***
**Ap226**	45	1005.453	0.000	***
**HB-C16-01**	276	4686.305	0.000	***
**A(B)124**	136	271.635	0.000	***
**AP019**	45	101.211	0.000	***
**Ap273**	6	13.536	0.035	*
**Ap289**	351	621.113	0.000	***
**HB-C16-05**	171	1834.959	0.000	***
**A043**	66	576.778	0.000	***
**Ap049**	28	262.995	0.000	***
**Ap288**	15	1583.362	0.000	***

Key: ns=not significant, * P<0.05, ** P<0.01, *** P<0.001

Table S3b: Summary of Chi-Square Tests for Hardy-Weinberg Equilibrium in samples from flowers

Locus	DF	ChiSq	Prob	Signif
**A(B)024**	6	5.810	0.445	ns
**A088**	45	29.489	0.964	ns
**AP043**	45	38.656	0.736	ns
**Ap113**	120	274.628	0.000	***
**Ap218**	10	109.131	0.000	***
**Ap249**	28	27.692	0.481	ns
**A007**	105	62.496	1.000	ns
**A014**	231	222.546	0.643	ns
**A079**	78	78.666	0.458	ns
**Ac306**	55	36.662	0.973	ns
**Ap068**	55	45.388	0.819	ns
**Ap223**	10	6.731	0.751	ns
**Ap226**	36	580.216	0.000	***
**HB-C16-01**	190	513.221	0.000	***
**A(B)124**	136	104.407	0.980	ns
**AP019**	21	15.983	0.771	ns
**Ap273**	6	10.420	0.108	ns
**Ap289**	276	270.963	0.574	ns
**HB-C16-05**	136	93.218	0.998	ns
**A043**	28	280.493	0.000	***
**Ap049**	15	86.859	0.000	***
**Ap288**	6	2.041	0.916	ns

Key: ns=not significant, * P<0.05, ** P<0.01, *** P<0.001
