# Supplementary material for: Assessing Genetic Diversity and Population Structure of Western Honey Bees in the Czech Republic Using 22 Microsatellite Loci
Source: Insects. 2025 Jan 9;16(1):55. doi: 10.3390/insects16010055 (PMC11766434; doi:10.3390/insects16010055)
Supplement: Supplementary file 1 [file insects-16-00055-s001.zip › Table S4 a-b.pdf]

Table S4a: Parameters of the genetic diversity of *Apis mellifera* in 77 districts collected from hives

Pop		N	Na	Ne	I	Ho	He	uHe	F
**BE**	Mean	44.909	6.591	3.049	1.212	0.537	0.576	0.583	0.056
	SE	0.063	0.663	0.457	0.106	0.036	0.040	0.040	0.022
**BI**	Mean	60.818	7.000	2.903	1.214	0.568	0.572	0.577	-0.007
	SE	0.107	0.635	0.346	0.098	0.034	0.037	0.038	0.023
**BK**	Mean	44.045	6.591	2.863	1.141	0.522	0.543	0.549	0.020
	SE	0.512	0.647	0.448	0.109	0.039	0.043	0.043	0.031
**BM**	Mean	27.182	5.500	2.797	1.125	0.541	0.548	0.559	-0.001
	SE	0.591	0.557	0.340	0.105	0.045	0.044	0.045	0.036
**BN**	Mean	51.000	6.818	2.733	1.171	0.545	0.558	0.564	0.023
	SE	0.000	0.683	0.344	0.089	0.037	0.035	0.035	0.029
**BR**	Mean	50.091	6.227	2.884	1.205	0.557	0.586	0.592	0.051
	SE	0.550	0.596	0.316	0.096	0.034	0.035	0.035	0.027
**BV**	Mean	71.727	6.773	2.827	1.169	0.565	0.564	0.568	-0.016
	SE	0.639	0.705	0.363	0.098	0.035	0.036	0.036	0.031
**CB**	Mean	56.545	6.591	2.765	1.158	0.528	0.554	0.559	0.034
	SE	0.171	0.650	0.332	0.098	0.034	0.038	0.038	0.024
**CK**	Mean	43.955	6.545	2.855	1.183	0.554	0.563	0.569	0.022
	SE	0.820	0.720	0.406	0.098	0.039	0.037	0.037	0.019
**CL**	Mean	44.000	6.545	2.693	1.178	0.537	0.571	0.578	0.038
	SE	0.830	0.627	0.240	0.088	0.035	0.034	0.034	0.037
**CR**	Mean	44.955	6.182	2.915	1.132	0.504	0.534	0.540	0.061
	SE	0.045	0.640	0.431	0.118	0.047	0.049	0.049	0.022
**CV**	Mean	44.000	6.409	2.878	1.220	0.572	0.589	0.596	0.039
	SE	0.335	0.670	0.305	0.092	0.035	0.032	0.033	0.028
**DC**	Mean	26.182	5.273	2.964	1.142	0.526	0.569	0.580	0.078
	SE	0.352	0.475	0.373	0.105	0.049	0.041	0.042	0.043
**DO**	Mean	42.273	6.136	2.584	1.104	0.545	0.545	0.552	-0.009
	SE	1.196	0.528	0.255	0.083	0.039	0.036	0.037	0.035
**FM**	Mean	44.227	6.682	2.925	1.213	0.561	0.577	0.583	0.015
	SE	0.441	0.713	0.363	0.102	0.037	0.040	0.041	0.024
**HB**	Mean	44.955	6.500	2.923	1.172	0.562	0.555	0.561	-0.006
	SE	0.045	0.689	0.414	0.109	0.047	0.043	0.044	0.021
**HK**	Mean	44.091	6.182	2.774	1.133	0.524	0.546	0.552	0.047
	SE	0.322	0.689	0.391	0.102	0.042	0.039	0.040	0.032
**HO**	Mean	49.318	7.136	2.907	1.225	0.572	0.581	0.587	0.018
	SE	0.274	0.854	0.377	0.094	0.034	0.034	0.034	0.021
**CH**	Mean	45.909	6.409	3.091	1.239	0.575	0.597	0.604	0.031
	SE	0.294	0.623	0.385	0.094	0.034	0.036	0.036	0.022
**JC**	Mean	44.727	6.909	2.981	1.205	0.566	0.566	0.572	-0.005
	SE	0.135	0.726	0.389	0.112	0.045	0.042	0.043	0.024
**JE**	Mean	44.273	6.591	2.790	1.184	0.547	0.573	0.579	0.034
	SE	0.288	0.692	0.322	0.096	0.035	0.036	0.036	0.029
**JH**	Mean	44.864	6.682	2.868	1.203	0.551	0.569	0.575	0.027
	SE	0.075	0.575	0.329	0.098	0.041	0.040	0.040	0.026
**JI**	Mean	44.182	6.500	2.672	1.123	0.522	0.536	0.542	0.026
	SE	0.521	0.602	0.326	0.101	0.042	0.041	0.042	0.021
**JN**	Mean	43.818	6.500	2.995	1.179	0.522	0.558	0.564	0.059
	SE	0.340	0.797	0.474	0.114	0.041	0.042	0.043	0.027
**KI**	Mean	44.318	6.727	3.013	1.214	0.546	0.578	0.585	0.037
	SE	0.413	0.724	0.415	0.099	0.030	0.037	0.037	0.028

**KH**	Mean	44.727	6.864	2.742	1.181	0.568	0.561	0.567	-0.014
	SE	0.117	0.736	0.297	0.095	0.040	0.038	0.039	0.022
**KD**	Mean	42.591	6.455	2.822	1.194	0.545	0.564	0.571	0.030
	SE	0.783	0.664	0.344	0.100	0.039	0.038	0.038	0.024
**KM**	Mean	74.545	7.182	2.719	1.173	0.518	0.557	0.561	0.052
	SE	0.409	0.723	0.325	0.093	0.039	0.037	0.037	0.039
**KO**	Mean	44.136	6.045	2.821	1.158	0.558	0.564	0.571	0.015
	SE	0.274	0.619	0.359	0.096	0.038	0.036	0.036	0.025
**KT**	Mean	43.318	6.409	2.993	1.233	0.587	0.592	0.599	0.005
	SE	0.771	0.591	0.350	0.095	0.036	0.036	0.037	0.023
**KV**	Mean	44.045	6.273	2.920	1.188	0.558	0.572	0.579	0.033
	SE	0.357	0.645	0.430	0.101	0.038	0.035	0.035	0.028
**LI**	Mean	42.591	6.955	2.882	1.241	0.555	0.583	0.590	0.035
	SE	0.726	0.725	0.314	0.093	0.031	0.034	0.035	0.026
**LN**	Mean	41.227	6.500	3.051	1.180	0.518	0.557	0.563	0.064
	SE	0.279	0.732	0.476	0.116	0.042	0.045	0.045	0.017
**LT**	Mean	41.227	6.773	3.110	1.268	0.585	0.606	0.613	0.027
	SE	0.366	0.596	0.392	0.090	0.032	0.032	0.032	0.027
**MB**	Mean	44.364	6.864	3.200	1.279	0.586	0.608	0.614	0.031
	SE	0.408	0.678	0.383	0.099	0.036	0.037	0.038	0.025
**ME**	Mean	43.182	6.182	2.890	1.172	0.529	0.561	0.568	0.050
	SE	1.143	0.650	0.404	0.104	0.039	0.040	0.041	0.033
**MO**	Mean	35.864	6.909	3.037	1.211	0.534	0.564	0.572	0.030
	SE	0.075	0.811	0.432	0.116	0.039	0.045	0.045	0.028
**NA**	Mean	54.727	6.864	2.826	1.205	0.561	0.581	0.586	0.030
	SE	0.527	0.662	0.286	0.092	0.039	0.035	0.036	0.031
**NB**	Mean	44.682	6.227	2.894	1.182	0.541	0.569	0.576	0.055
	SE	0.166	0.613	0.376	0.098	0.040	0.038	0.038	0.026
**NJ**	Mean	49.045	6.227	2.709	1.139	0.543	0.550	0.556	0.003
	SE	0.507	0.542	0.313	0.092	0.038	0.038	0.038	0.032
**OC**	Mean	44.864	6.455	2.838	1.141	0.531	0.537	0.543	-0.005
	SE	0.075	0.717	0.466	0.107	0.038	0.041	0.041	0.026
**OP**	Mean	67.000	6.636	2.879	1.170	0.509	0.557	0.561	0.066
	SE	1.122	0.633	0.383	0.105	0.037	0.040	0.041	0.032
**OV**	Mean	44.636	6.591	3.062	1.229	0.564	0.579	0.586	0.023
	SE	0.181	0.640	0.387	0.108	0.040	0.042	0.042	0.022
**PU**	Mean	43.818	7.000	2.902	1.191	0.543	0.557	0.564	0.019
	SE	0.486	0.770	0.419	0.108	0.041	0.040	0.041	0.032
**PB**	Mean	61.909	6.955	2.953	1.237	0.565	0.590	0.595	0.043
	SE	0.469	0.656	0.339	0.094	0.037	0.035	0.035	0.028
**PE**	Mean	50.227	6.818	2.915	1.188	0.558	0.563	0.569	0.015
	SE	0.522	0.643	0.424	0.102	0.043	0.040	0.040	0.027
**PY**	Mean	46.318	6.636	2.778	1.154	0.526	0.550	0.556	0.051
	SE	0.191	0.682	0.384	0.103	0.040	0.038	0.038	0.020
**PHA**	Mean	42.545	5.909	2.845	1.144	0.555	0.548	0.554	-0.022
	SE	0.157	0.610	0.380	0.110	0.042	0.042	0.042	0.024
**PI**	Mean	50.045	6.500	2.898	1.162	0.534	0.564	0.569	0.039
	SE	0.326	0.647	0.435	0.099	0.033	0.037	0.037	0.022
**PJ**	Mean	44.545	6.318	3.012	1.203	0.566	0.579	0.586	0.034
	SE	0.261	0.659	0.434	0.103	0.040	0.038	0.039	0.023
**PM**	Mean	34.864	5.636	2.746	1.139	0.555	0.566	0.574	0.014
	SE	0.136	0.605	0.303	0.089	0.035	0.035	0.035	0.026

**PR**	Mean	44.273	6.864	2.797	1.217	0.572	0.578	0.584	-0.003
	SE	0.330	0.675	0.293	0.090	0.035	0.035	0.035	0.026
**PS**	Mean	38.273	6.409	2.953	1.202	0.565	0.583	0.591	0.019
	SE	0.662	0.545	0.341	0.095	0.038	0.037	0.038	0.037
**PT**	Mean	60.455	6.909	3.029	1.222	0.570	0.577	0.582	0.011
	SE	0.772	0.714	0.399	0.105	0.039	0.039	0.040	0.026
**PV**	Mean	44.909	6.500	2.791	1.160	0.533	0.555	0.561	0.040
	SE	0.063	0.651	0.380	0.096	0.039	0.037	0.037	0.026
**PZ**	Mean	40.682	6.455	2.957	1.203	0.544	0.569	0.576	0.039
	SE	0.691	0.606	0.359	0.103	0.044	0.041	0.042	0.035
**RA**	Mean	31.091	5.864	2.732	1.140	0.526	0.550	0.559	0.051
	SE	0.271	0.604	0.300	0.099	0.044	0.040	0.041	0.033
**RK**	Mean	73.409	6.818	2.752	1.207	0.553	0.578	0.582	0.032
	SE	1.062	0.720	0.265	0.091	0.032	0.033	0.033	0.029
**RO**	Mean	43.182	6.227	2.691	1.159	0.513	0.562	0.568	0.080
	SE	0.761	0.588	0.253	0.094	0.036	0.039	0.039	0.024
**SM**	Mean	44.545	6.455	2.641	1.108	0.505	0.530	0.536	0.033
	SE	0.300	0.660	0.339	0.101	0.039	0.042	0.043	0.024
**SO**	Mean	44.955	6.636	2.853	1.198	0.548	0.574	0.580	0.043
	SE	0.045	0.619	0.327	0.093	0.040	0.038	0.038	0.026
**ST**	Mean	68.455	7.273	2.925	1.248	0.584	0.589	0.593	-0.002
	SE	0.409	0.748	0.351	0.094	0.031	0.032	0.033	0.027
**SU**	Mean	43.500	6.318	2.866	1.199	0.575	0.582	0.589	0.008
	SE	1.087	0.567	0.343	0.086	0.034	0.032	0.032	0.028
**SY**	Mean	43.955	6.318	2.767	1.147	0.531	0.554	0.561	0.030
	SE	0.408	0.646	0.335	0.099	0.038	0.039	0.040	0.028
**TA**	Mean	49.591	6.273	2.696	1.136	0.493	0.546	0.551	0.075
	SE	0.657	0.567	0.302	0.097	0.036	0.041	0.041	0.034
**TC**	Mean	36.909	5.909	2.724	1.142	0.514	0.556	0.564	0.047
	SE	0.759	0.538	0.283	0.092	0.032	0.039	0.039	0.034
**TP**	Mean	35.818	6.545	2.773	1.172	0.531	0.558	0.566	0.045
	SE	0.142	0.640	0.351	0.095	0.036	0.036	0.036	0.029
**TR**	Mean	43.864	6.455	2.939	1.172	0.572	0.561	0.567	-0.014
	SE	0.136	0.613	0.421	0.103	0.046	0.042	0.042	0.026
**TU**	Mean	36.000	5.909	2.820	1.167	0.576	0.566	0.574	-0.012
	SE	0.000	0.599	0.346	0.099	0.043	0.038	0.038	0.025
**UH**	Mean	45.000	6.136	2.815	1.128	0.560	0.545	0.552	-0.037
	SE	0.000	0.622	0.412	0.104	0.040	0.042	0.042	0.021
**UL**	Mean	43.909	6.682	2.756	1.172	0.565	0.560	0.567	-0.010
	SE	0.112	0.698	0.335	0.092	0.038	0.036	0.037	0.017
**UO**	Mean	44.182	6.364	2.815	1.177	0.538	0.563	0.569	0.038
	SE	0.370	0.609	0.325	0.095	0.037	0.037	0.037	0.026
**VS**	Mean	44.364	6.818	2.949	1.224	0.564	0.579	0.586	0.021
	SE	0.291	0.701	0.347	0.100	0.042	0.041	0.041	0.026
**VY**	Mean	43.909	6.500	2.735	1.130	0.536	0.540	0.547	0.050
	SE	0.278	0.627	0.369	0.101	0.046	0.040	0.040	0.054
**ZL**	Mean	43.864	6.591	2.859	1.204	0.563	0.574	0.580	0.010
	SE	0.231	0.644	0.358	0.093	0.032	0.035	0.035	0.022
**ZN**	Mean	43.955	5.909	2.733	1.113	0.522	0.535	0.542	0.017
	SE	0.477	0.573	0.360	0.100	0.043	0.040	0.041	0.035
**ZR**	Mean	74.545	6.818	3.022	1.203	0.562	0.576	0.580	0.021
	SE	0.365	0.660	0.467	0.101	0.036	0.035	0.035	0.032

		N	Na	Ne	I	Ho	He	uHe	F
**Total**	Mean	46.429	6.495	2.863	1.181	0.547	0.565	0.572	0.026
	SE	0.234	0.073	0.041	0.011	0.004	0.004	0.004	0.003

N, number of samples; Na, No. of different alleles; Ne, No. of effective alleles = 1 / (Sum p_i^2); I, Shannon's information index = -1* Sum (p_i * Ln (p_i)); Ho, observed heterozygosity = No. of Hets / N; He, expected heterozygosity = 1 - Sum p_i^2; uHe, unbiased expected heterozygosity = (2N / (2N-1)) * He; F, fixation index = (He - Ho) / He = 1 - (Ho / He); where p_i is the frequency of the ith allele for the population

Abbreviation and name of district: BE Beroun; BI Brno-venkov; BK Blansko; BM Brno-město; BN Benešov; BR Bruntál; BV Břeclav; CB České Budějovice; CK Český Krumlov; CL Česká Lípa; CR Chrudim; CV Chomutov; DC Děčín; DO Domažlice; FM Frýdek Místek; HB Havlíčkův Brod; HK Hradec Králové; HO Hodonín; CH Cheb; JC Jičín; JE Jeseník; JH Jindřichův Hradec; JI Jihlava; JN Jablonec nad Nisou; KI Karviná; KH Kutná Hora; KD Kladno; KM Kroměříž; KO Kolín; KT Klatovy; KV Karlovy Vary; LI Liberec; LN Louny; LT Litoměřice; MB Mladá Boleslav; ME Mělník; MO Most; NA Náchod; NB Nymburk; NJ Nový Jičín; OC Olomouc; OP Opava; OV Ostrava-město; PU Pardubice; PB Příbram; PE Pelhřimov; PY Praha-východ; PHA Praha; PI Písek; PJ Plzeň-jih; PM Plzeň-město; PR Přerov; PS Plzeň-sever; PT Prachatice; PV Prostějov; PZ Praha-západ; RA Rakovník; RK Rychnov nad Kněžnou; RO Rokycany; SM Semily; SO Sokolov; ST Strakonice ; SU Šumperk; SY Svitavy; TA Tábor; TC Tachov; TP Teplice; TR Třebíč; TU Trutnov; UH Uherské Hradiště; UL Ústí nad Labem; UO Ústí nad Orlicí; VS Vsetín; VY Vyškov; ZL Zlín; ZN Znojmo; ZR Žďár nad Sázavou

Table S4b: Parameters of the genetic diversity of *Apis mellifera* in 77 districts collected from flowers

Pop		N	Na	Ne	I	Ho	He	uHe	F
**BE**	Mean	10.864	4.500	2.675	1.040	0.544	0.527	0.553	-0.051
	SE	0.100	0.404	0.305	0.105	0.046	0.045	0.048	0.040
**BI**	Mean	4.864	3.182	2.264	0.879	0.525	0.494	0.551	-0.092
	SE	0.075	0.182	0.180	0.075	0.052	0.041	0.046	0.061
**BK**	Mean	6.000	3.364	2.352	0.890	0.523	0.485	0.530	-0.104
	SE	0.000	0.319	0.250	0.098	0.059	0.047	0.051	0.067
**BM**	Mean	6.955	3.818	2.479	0.963	0.588	0.513	0.553	-0.149
	SE	0.045	0.364	0.296	0.092	0.046	0.038	0.041	0.039
**BN**	Mean	7.909	4.227	2.604	1.041	0.559	0.537	0.573	-0.040
	SE	0.091	0.366	0.263	0.095	0.053	0.042	0.045	0.049
**BR**	Mean	4.909	3.636	2.711	0.987	0.541	0.522	0.580	-0.041
	SE	0.063	0.339	0.305	0.111	0.065	0.052	0.058	0.076
**BV**	Mean	9.909	4.773	2.797	1.112	0.602	0.553	0.583	-0.095
	SE	0.063	0.400	0.312	0.100	0.055	0.044	0.046	0.048
**CB**	Mean	10.000	4.273	2.603	1.000	0.523	0.512	0.539	-0.036
	SE	0.000	0.401	0.328	0.104	0.048	0.048	0.050	0.031
**CK**	Mean	9.909	3.909	2.436	0.974	0.544	0.514	0.542	-0.080
	SE	0.063	0.294	0.223	0.086	0.044	0.042	0.045	0.035
**CL**	Mean	6.000	3.818	2.619	1.003	0.583	0.530	0.578	-0.096
	SE	0.000	0.352	0.258	0.102	0.058	0.046	0.050	0.060
**CR**	Mean	6.000	3.591	2.384	0.925	0.485	0.496	0.541	0.033
	SE	0.000	0.320	0.247	0.093	0.057	0.044	0.048	0.062
**CV**	Mean	6.000	3.727	2.725	0.998	0.561	0.534	0.583	-0.062
	SE	0.000	0.379	0.322	0.105	0.060	0.045	0.049	0.067
**DC**	Mean	5.727	3.364	2.309	0.914	0.506	0.506	0.556	0.014
	SE	0.135	0.214	0.173	0.080	0.060	0.041	0.046	0.063
**DO**	Mean	6.000	3.636	2.421	0.963	0.545	0.521	0.568	-0.054
	SE	0.000	0.283	0.221	0.086	0.047	0.039	0.043	0.043
**FM**	Mean	5.955	3.818	2.640	1.017	0.555	0.541	0.590	-0.021
	SE	0.045	0.299	0.302	0.089	0.051	0.038	0.042	0.063
**HB**	Mean	5.955	3.636	2.602	0.950	0.541	0.502	0.548	-0.097
	SE	0.045	0.381	0.298	0.115	0.060	0.054	0.059	0.048
**HK**	Mean	6.000	3.409	2.139	0.862	0.500	0.466	0.508	-0.079
	SE	0.000	0.204	0.174	0.079	0.051	0.042	0.046	0.041
**HO**	Mean	9.955	4.409	2.378	1.017	0.517	0.523	0.551	0.041
	SE	0.045	0.398	0.175	0.087	0.046	0.039	0.041	0.059
**CH**	Mean	7.773	3.955	2.519	1.019	0.554	0.544	0.583	-0.018
	SE	0.185	0.332	0.216	0.084	0.055	0.036	0.039	0.067
**JC**	Mean	6.000	3.545	2.442	0.907	0.515	0.482	0.526	-0.078
	SE	0.000	0.376	0.302	0.107	0.060	0.049	0.054	0.057
**JE**	Mean	6.000	3.773	2.517	0.993	0.644	0.528	0.576	-0.222
	SE	0.000	0.341	0.263	0.093	0.054	0.040	0.043	0.048
**JH**	Mean	9.864	4.409	2.573	1.049	0.573	0.533	0.562	-0.090
	SE	0.136	0.333	0.281	0.089	0.042	0.039	0.042	0.034
**JI**	Mean	5.955	3.227	2.271	0.856	0.523	0.473	0.517	-0.095
	SE	0.045	0.263	0.252	0.088	0.061	0.043	0.047	0.059
**JN**	Mean	5.500	3.773	2.644	1.023	0.641	0.545	0.600	-0.190
	SE	0.109	0.322	0.262	0.094	0.053	0.043	0.047	0.050
**KI**	Mean	6.000	3.545	2.468	0.938	0.515	0.503	0.549	-0.035
	SE	0.000	0.285	0.253	0.097	0.054	0.047	0.051	0.052
**KH**	Mean	6.909	3.864	2.721	1.035	0.700	0.554	0.597	-0.266
	SE	0.063	0.337	0.270	0.097	0.057	0.042	0.045	0.047
**KD**	Mean	6.000	3.955	2.778	1.025	0.545	0.533	0.581	-0.054

	SE	0.000	0.397	0.364	0.109	0.044	0.046	0.051	0.047
**KM**	Mean	14.818	5.182	2.763	1.129	0.534	0.555	0.575	0.019
	SE	0.107	0.435	0.329	0.094	0.038	0.038	0.040	0.038
**KO**	Mean	6.000	3.591	2.424	0.946	0.508	0.513	0.559	0.003
	SE	0.000	0.299	0.216	0.092	0.061	0.044	0.048	0.073
**KT**	Mean	6.000	3.682	2.516	0.971	0.583	0.521	0.568	-0.114
	SE	0.000	0.318	0.272	0.092	0.059	0.042	0.046	0.056
**KV**	Mean	6.000	3.636	2.592	1.025	0.591	0.564	0.615	-0.074
	SE	0.000	0.298	0.201	0.080	0.038	0.034	0.037	0.050
**LI**	Mean	6.000	4.000	2.544	1.033	0.591	0.540	0.589	-0.079
	SE	0.000	0.316	0.262	0.084	0.058	0.035	0.038	0.065
**LN**	Mean	6.000	3.955	2.722	1.057	0.576	0.565	0.616	-0.024
	SE	0.000	0.339	0.284	0.088	0.045	0.035	0.038	0.052
**LT**	Mean	6.000	3.591	2.614	0.949	0.530	0.516	0.563	-0.017
	SE	0.000	0.425	0.334	0.108	0.060	0.045	0.049	0.067
**MB**	Mean	5.909	3.818	2.580	1.014	0.532	0.536	0.586	0.000
	SE	0.063	0.333	0.244	0.095	0.052	0.044	0.048	0.048
**ME**	Mean	6.000	3.727	2.787	1.025	0.636	0.556	0.607	-0.144
	SE	0.000	0.396	0.334	0.101	0.054	0.040	0.043	0.053
**MO**	Mean	6.000	3.955	2.762	1.065	0.659	0.564	0.615	-0.185
	SE	0.000	0.369	0.280	0.097	0.053	0.041	0.045	0.058
**NA**	Mean	6.000	3.818	2.630	1.030	0.553	0.550	0.600	0.007
	SE	0.000	0.276	0.234	0.087	0.055	0.043	0.047	0.051
**NB**	Mean	6.000	3.636	2.578	0.974	0.591	0.518	0.565	-0.111
	SE	0.000	0.358	0.280	0.105	0.069	0.050	0.054	0.073
**NJ**	Mean	6.000	4.000	2.858	1.045	0.614	0.549	0.598	-0.114
	SE	0.000	0.411	0.395	0.105	0.054	0.044	0.048	0.044
**OC**	Mean	14.773	5.273	2.878	1.162	0.561	0.578	0.598	0.021
	SE	0.130	0.515	0.316	0.097	0.039	0.037	0.039	0.044
**OP**	Mean	6.000	3.636	2.435	0.954	0.515	0.513	0.559	0.033
	SE	0.000	0.326	0.235	0.092	0.061	0.041	0.045	0.077
**OV**	Mean	6.000	3.545	2.595	0.982	0.545	0.538	0.587	-0.032
	SE	0.000	0.320	0.244	0.096	0.052	0.044	0.048	0.054
**PU**	Mean	5.955	3.545	2.347	0.923	0.464	0.497	0.542	0.040
	SE	0.045	0.292	0.228	0.091	0.053	0.042	0.045	0.069
**PB**	Mean	5.000	3.727	2.680	1.037	0.545	0.559	0.621	0.033
	SE	0.000	0.281	0.246	0.086	0.056	0.038	0.042	0.078
**PE**	Mean	5.864	3.591	2.584	1.017	0.511	0.563	0.616	0.070
	SE	0.075	0.269	0.212	0.074	0.054	0.032	0.035	0.082
**PHA**	Mean	6.000	3.636	2.611	0.971	0.553	0.521	0.569	-0.058
	SE	0.000	0.352	0.303	0.101	0.058	0.046	0.050	0.058
**PY**	Mean	6.000	4.182	2.871	1.124	0.598	0.583	0.636	-0.035
	SE	0.000	0.346	0.247	0.094	0.054	0.041	0.045	0.054
**PI**	Mean	6.000	3.909	2.829	1.096	0.644	0.587	0.640	-0.071
	SE	0.000	0.301	0.270	0.081	0.055	0.033	0.036	0.074
**PJ**	Mean	6.636	3.955	2.600	1.005	0.518	0.522	0.565	-0.027
	SE	0.155	0.319	0.289	0.098	0.047	0.045	0.049	0.049
**PM**	Mean	9.773	4.455	2.685	1.104	0.566	0.568	0.599	-0.002
	SE	0.146	0.314	0.250	0.079	0.043	0.034	0.036	0.049
**PR**	Mean	5.000	3.455	2.506	0.942	0.545	0.511	0.568	-0.093
	SE	0.000	0.314	0.257	0.100	0.055	0.047	0.052	0.057
**PS**	Mean	7.909	3.909	2.473	0.998	0.516	0.527	0.563	0.031
	SE	0.063	0.315	0.220	0.088	0.054	0.041	0.043	0.053
**PT**	Mean	5.864	3.682	2.537	0.989	0.556	0.529	0.579	-0.034
	SE	0.075	0.274	0.246	0.087	0.070	0.043	0.047	0.083
**PV**	Mean	6.000	4.000	2.412	1.004	0.576	0.523	0.570	-0.084

	SE	0.000	0.287	0.221	0.081	0.052	0.036	0.039	0.070
**PZ**	Mean	5.909	3.682	2.408	0.944	0.568	0.505	0.552	-0.141
	SE	0.063	0.325	0.254	0.090	0.050	0.040	0.044	0.062
**RA**	Mean	5.182	3.500	2.498	0.961	0.568	0.523	0.579	-0.080
	SE	0.084	0.285	0.223	0.094	0.066	0.047	0.052	0.064
**RK**	Mean	6.000	3.682	2.451	0.992	0.591	0.537	0.586	-0.077
	SE	0.000	0.282	0.207	0.079	0.057	0.034	0.037	0.080
**RO**	Mean	6.000	3.545	2.425	0.933	0.568	0.501	0.546	-0.125
	SE	0.000	0.285	0.275	0.090	0.054	0.041	0.045	0.047
**SM**	Mean	5.955	3.818	2.611	1.029	0.567	0.547	0.598	-0.027
	SE	0.045	0.306	0.242	0.088	0.055	0.038	0.042	0.062
**SO**	Mean	6.000	3.727	2.628	0.994	0.538	0.531	0.579	-0.013
	SE	0.000	0.317	0.301	0.097	0.062	0.043	0.047	0.076
**ST**	Mean	6.000	4.091	2.821	1.128	0.674	0.600	0.655	-0.136
	SE	0.000	0.328	0.223	0.079	0.035	0.029	0.032	0.043
**SU**	Mean	5.955	3.682	2.423	0.949	0.535	0.506	0.552	-0.071
	SE	0.045	0.282	0.254	0.086	0.052	0.041	0.045	0.052
**SY**	Mean	6.000	3.682	2.501	0.972	0.485	0.524	0.572	0.078
	SE	0.000	0.318	0.284	0.089	0.050	0.038	0.041	0.075
**TA**	Mean	5.000	3.364	2.393	0.930	0.482	0.512	0.569	0.015
	SE	0.000	0.233	0.215	0.085	0.047	0.043	0.048	0.072
**TC**	Mean	6.682	3.545	2.462	0.955	0.517	0.520	0.565	0.021
	SE	0.325	0.277	0.233	0.089	0.067	0.043	0.048	0.088
**TP**	Mean	6.000	3.682	2.593	1.001	0.606	0.544	0.593	-0.117
	SE	0.000	0.338	0.246	0.090	0.052	0.039	0.042	0.049
**TR**	Mean	9.955	4.091	2.497	1.001	0.585	0.524	0.552	-0.112
	SE	0.045	0.315	0.239	0.085	0.051	0.040	0.042	0.048
**TU**	Mean	5.864	3.818	2.631	1.053	0.556	0.567	0.620	0.003
	SE	0.075	0.260	0.225	0.074	0.040	0.032	0.035	0.050
**UH**	Mean	14.955	4.591	2.484	0.999	0.539	0.511	0.528	-0.057
	SE	0.045	0.430	0.278	0.097	0.047	0.043	0.044	0.035
**UL**	Mean	5.727	3.591	2.496	0.954	0.571	0.514	0.563	-0.081
	SE	0.097	0.370	0.247	0.102	0.065	0.047	0.052	0.069
**UO**	Mean	6.000	3.545	2.339	0.928	0.553	0.503	0.548	-0.099
	SE	0.000	0.244	0.219	0.080	0.053	0.040	0.043	0.051
**VS**	Mean	15.000	5.364	2.915	1.174	0.536	0.574	0.594	0.053
	SE	0.000	0.449	0.364	0.097	0.039	0.039	0.041	0.037
**VY**	Mean	7.864	3.864	2.551	1.010	0.595	0.536	0.573	-0.121
	SE	0.100	0.274	0.243	0.087	0.051	0.041	0.044	0.046
**ZL**	Mean	9.955	4.409	2.608	1.038	0.530	0.530	0.558	0.011
	SE	0.045	0.387	0.284	0.100	0.052	0.045	0.047	0.034
**ZN**	Mean	9.909	4.364	2.673	1.026	0.589	0.523	0.551	-0.133
	SE	0.091	0.408	0.335	0.105	0.053	0.046	0.048	0.036
**ZR**	Mean	9.727	4.182	2.580	1.024	0.478	0.538	0.569	0.122
	SE	0.230	0.333	0.268	0.088	0.046	0.037	0.040	0.053

		**N**	**Na**	**Ne**	**I**	**Ho**	**He**	**uHe**	**F**
**Total**	Mean	7.066	3.871	2.565	0.999	0.557	0.530	0.574	-0.054
	SE	0.058	0.039	0.030	0.010	0.006	0.005	0.005	0.007

N, number of samples; Na, No. of different alleles; Ne, No. of effective alleles = 1 / (Sum p_i^2); I, Shannon's information index = -1* Sum (p_i * Ln (p_i)); Ho, observed heterozygosity = No. of Hets / N; He, expected heterozygosity = 1 - Sum p_i^2; uHe, unbiased expected heterozygosity = (2N / (2N-1)) * He; F, fixation index = (He - Ho) / He = 1 - (Ho / He); where p_i is the frequency of the ith allele for the population

Abbreviation and name of district: BE Beroun; BI Brno-venkov; BK Blansko; BM Brno-město; BN Benešov; BR Bruntál; BV Břeclav; CB České Budějovice; CK Český Krumlov; CL Česká Lípa; CR Chrudim; CV Chomutov; DC Děčín; DO Domažlice; FM Frýdek Místek; HB Havlíčkův Brod; HK Hradec Králové; HO Hodonín; CH Cheb; JC Jičín; JE Jeseník; JH Jindřichův Hradec; JI Jihlava; JN Jablonec nad Nisou; KI Karviná; KH Kutná Hora; KD Kladno; KM Kroměříž; KO Kolín; KT Klatovy; KV Karlovy Vary; LI Liberec; LN Louny; LT Litoměřice; MB Mladá Boleslav; ME Mělník; MO Most; NA Náchod; NB Nymburk; NJ Nový Jičín; OC Olomouc; OP Opava; OV Ostrava-město; PU Pardubice; PB Příbram; PE Pelhřimov; PY Praha-východ; PHA Praha; PI Písek; PJ Plzeň-jih; PM Plzeň-město; PR Přerov; PS Plzeň-sever; PT Prachatice; PV Prostějov; PZ Praha-západ; RA Rakovník; RK Rychnov nad Kněžnou; RO Rokycany; SM Semily; SO Sokolov; ST Strakonice ; SU Šumperk; SY Svitavy; TA Tábor; TC Tachov; TP Teplice; TR Třebíč; TU Trutnov; UH Uherské Hradiště; UL Ústí nad Labem; UO Ústí nad Orlicí; VS Vsetín; VY Vyškov; ZL Zlín; ZN Znojmo; ZR Žďár nad Sázavou
