# Supplementary material for: Assessing Genetic Diversity and Population Structure of Western Honey Bees in the Czech Republic Using 22 Microsatellite Loci
Source: Insects. 2025 Jan 9;16(1):55. doi: 10.3390/insects16010055 (PMC11766434; doi:10.3390/insects16010055)
Supplement: Supplementary file 1 [file insects-16-00055-s001.zip › Table S5 a-b.pdf]

Table S5a: Summary of private alleles by populations collected from hives

Pop	Locus	Allele	Freq
**BI**	A(B)024	104	0.008
**BR**	A079	92	0.010
**BV**	A043	141	0.007
**CR**	Ap223	164	0.011
**CV**	Ap249	212	0.022
**FM**	A079	118	0.014
**JC**	A007	106	0.022
**JI**	A079	116	0.022
**JI**	Ap226	240	0.011
**KT**	A088	136	0.011
**KT**	A088	145	0.022
**LI**	Ac306	178	0.011
**LT**	Ap049	136	0.012
**MB**	Ap289	189	0.011
**NJ**	A(B)024	99	0.020
**NJ**	Ap226	242	0.010
**OP**	Ap068	167	0.007
**OP**	A043	122	0.015
**OV**	A088	142	0.022
**PI**	Ap249	220	0.010
**PT**	A079	120	0.016
**PT**	A043	131	0.017
**PV**	Ap249	219	0.011
**PZ**	AP019	146	0.012
**SM**	Ap289	194	0.011
**ST**	A088	143	0.022
**TC**	AP043	132	0.013
**UH**	A007	138	0.011
**UH**	Ac306	179	0.011
**UH**	Ap289	196	0.022
**UL**	Ap288	122	0.011
**UO**	AP043	164	0.011
**VY**	Ap113	215	0.011
**VY**	Ap249	229	0.033

Abbreviation and name of district: BE Beroun; BI Brno-venkov; BK Blansko; BM Brno-město; BN Benešov; BR Bruntál; BV Břeclav; CB České Budějovice; CK Český Krumlov; CL Česká Lípa; CR Chrudim; CV Chomutov; DC Děčín; DO Domažlice; FM Frýdek Místek; HB Havlíčkův Brod; HK Hradec Králové; HO Hodonín; CH Cheb; JC Jičín; JE Jeseník; JH Jindřichův Hradec; JI Jihlava; JN Jablonec nad Nisou; KI Karviná; KH Kutná Hora; KD Kladno; KM Kroměříž; KO Kolín; KT Klatovy; KV Karlovy Vary; LI Liberec; LN Louny; LT Litoměřice; MB Mladá Boleslav; ME Mělník; MO Most; NA Náchod; NB Nymburk; NJ Nový Jičín; OC Olomouc; OP Opava; OV Ostrava-město; PU Pardubice; PB Příbram; PE Pelhřimov; PY Praha-východ; PHA Praha; PI Písek; PJ Plzeň-jih; PM Plzeň-město; PR Přerov; PS Plzeň-sever; PT Prachatice; PV Prostějov; PZ Praha-západ; RA Rakovník; RK Rychnov nad Kněžnou; RO Rokycany; SM Semily; SO Sokolov; ST Strakonice ; SU Šumperk; SY Svitavy; TA Tábor; TC Tachov; TP Teplice; TR Třebíč; TU Trutnov; UH Uherské Hradiště; UL Ústí nad Labem; UO Ústí nad Orlicí; VS Vsetín; VY Vyškov; ZL Zlín; ZN Znojmo; ZR Žďár nad Sázavou

Table S5b: Summary of private alleles by population collected from flowers

Pop	Locus	Allele	Freq
**BE**	Ap218	138	0.045
**BK**	A014	235	0.083
**CK**	A079	124	0.050
**CR**	Ap249	208	0.083
**CV**	HB-C16-01	303	0.083
**HO**	Ap249	219	0.050
**CH**	A088	130	0.063
**CH**	AP043	152	0.063
**CH**	A014	221	0.063
**JE**	Ap249	222	0.083
**JE**	Ap289	213	0.167
**JH**	A014	223	0.050
**KH**	Ap289	206	0.214
**KD**	A(B)124	238	0.083
**KM**	Ap068	169	0.033
**KM**	Ap289	217	0.033
**KO**	A007	132	0.083
**MO**	A007	111	0.083
**OC**	A014	242	0.033
**OC**	Ac306	163	0.033
**PU**	Ap289	202	0.083
**PR**	Ap289	191	0.100
**PT**	Ap288	128	0.083
**RO**	Ap289	219	0.083
**ST**	Ap223	180	0.083
**TC**	Ap226	228	0.143
**TP**	A014	244	0.083
**TP**	A(B)124	210	0.083
**TP**	A(B)124	242	0.083
**UH**	Ap249	212	0.033
**UL**	A014	236	0.083
**UO**	A043	141	0.083
**VS**	A(B)024	104	0.033
**VS**	Ac306	169	0.033
**VS**	A(B)124	240	0.033
**VY**	HB-C16-05	82	0.063
**ZL**	Ac306	157	0.050
**ZN**	HB-C16-05	80	0.100

Abbreviation and name of district: BE Beroun; BI Brno-venkov; BK Blansko; BM Brno-město; BN Benešov; BR Bruntál; BV Břeclav; CB České Budějovice; CK Český Krumlov; CL Česká Lípa; CR Chrudim; CV Chomutov; DC Děčín; DO Domažlice; FM Frýdek Místek; HB Havlíčkův Brod; HK Hradec Králové; HO Hodonín; CH Cheb; JC Jičín; JE Jeseník; JH Jindřichův Hradec; JI Jihlava; JN Jablonec nad Nisou; KI Karviná; KH Kutná Hora; KD Kladno; KM Kroměříž; KO Kolín; KT Klatovy; KV Karlovy Vary; LI Liberec; LN Louny; LT Litoměřice; MB Mladá Boleslav; ME Mělník; MO Most; NA Náchod; NB Nymburk; NJ Nový Jičín; OC Olomouc; OP Opava; OV Ostrava-město; PU Pardubice; PB Příbram; PE Pelhřimov; PY Praha-východ; PHA Praha; PI Písek; PJ Plzeň-jih; PM Plzeň-město; PR Přerov; PS Plzeň-sever; PT Prachatice; PV Prostějov; PZ Praha-západ; RA Rakovník; RK Rychnov nad Kněžnou; RO Rokycany; SM Semily; SO Sokolov; ST Strakonice ; SU Šumperk; SY Svitavy; TA Tábor; TC Tachov; TP Teplice; TR Třebíč; TU Trutnov; UH Uherské Hradiště; UL Ústí nad Labem; UO Ústí nad Orlicí; VS Vsetín; VY Vyškov; ZL Zlín; ZN Znojmo; ZR Žďár nad Sázavou
