# Supplementary material for: Assessing Genetic Diversity and Population Structure of Western Honey Bees in the Czech Republic Using 22 Microsatellite Loci
Source: Insects. 2025 Jan 9;16(1):55. doi: 10.3390/insects16010055 (PMC11766434; doi:10.3390/insects16010055)
Supplement: Supplementary file 1 [file insects-16-00055-s001.zip › Table S8 a-b.pdf]

Table S8a: Detailed results from Structure analysis – populations from hives

Proportion of membership of each pre-defined population in each of the 3 clusters:

Given Pop	Inferred Clusters 1	2	3	Number of Individuals
1:	0.554	0.210	0.236	45
2:	0.485	0.290	0.226	61
3:	0.512	0.186	0.302	45
4:	0.450	0.163	0.387	28
5:	0.431	0.210	0.359	51
6:	0.441	0.293	0.266	51
7:	0.534	0.158	0.308	73
8:	0.595	0.150	0.255	57
9:	0.375	0.157	0.468	45
10:	0.378	0.296	0.326	45
11:	0.405	0.189	0.406	45
12:	0.365	0.286	0.349	45
13:	0.643	0.248	0.110	27
14:	0.451	0.140	0.409	45
15:	0.393	0.211	0.396	45
16:	0.332	0.236	0.432	45
17:	0.318	0.329	0.352	45
18:	0.312	0.254	0.434	50
19:	0.322	0.329	0.349	47
20:	0.369	0.328	0.302	45
21:	0.411	0.277	0.312	45
22:	0.374	0.326	0.301	45
23:	0.531	0.216	0.253	45
24:	0.387	0.299	0.313	45
25:	0.354	0.225	0.421	45
26:	0.430	0.203	0.367	45
27:	0.276	0.546	0.178	44
28:	0.453	0.270	0.277	75
29:	0.365	0.120	0.515	45
30:	0.506	0.142	0.352	45
31:	0.548	0.187	0.265	45
32:	0.556	0.167	0.277	45
33:	0.395	0.241	0.364	42
34:	0.424	0.321	0.255	42
35:	0.361	0.268	0.371	45
36:	0.439	0.288	0.273	45
37:	0.427	0.248	0.325	36
38:	0.398	0.353	0.249	56
39:	0.459	0.173	0.368	45
40:	0.586	0.173	0.242	51
41:	0.448	0.267	0.285	45
42:	0.568	0.228	0.204	69
43:	0.429	0.302	0.269	45
44:	0.466	0.271	0.263	45
45:	0.235	0.294	0.472	63
46:	0.456	0.281	0.262	51
47:	0.451	0.276	0.273	45
48:	0.632	0.114	0.255	45
49:	0.454	0.167	0.380	51
50:	0.374	0.321	0.305	45
51:	0.530	0.184	0.286	36
52:	0.404	0.249	0.348	45
53:	0.460	0.204	0.336	42
54:	0.364	0.431	0.205	62
55:	0.460	0.275	0.265	45
56:	0.457	0.274	0.269	45
57:	0.397	0.271	0.332	32
58:	0.432	0.204	0.363	75
59:	0.262	0.327	0.411	45
60:	0.437	0.205	0.359	45
61:	0.442	0.295	0.262	45
62:	0.461	0.309	0.230	69
63:	0.451	0.138	0.411	45
64:	0.411	0.403	0.186	45

65:	0.433	0.202	0.365	51
66:	0.300	0.300	0.400	39
67:	0.335	0.412	0.254	36
68:	0.377	0.220	0.403	44
69:	0.331	0.385	0.284	36
70:	0.503	0.202	0.294	45
71:	0.293	0.455	0.252	45
72:	0.370	0.337	0.292	45
73:	0.273	0.226	0.501	45
74:	0.515	0.162	0.323	45
75:	0.608	0.177	0.216	45
76:	0.403	0.276	0.321	45
77:	0.409	0.277	0.315	75

Names of population-district are in Supplement Table S1.

Allele-freq. divergence among pops (Net nucleotide distance), computed using point estimates of P.

	1	2	3
1	-	0.0226	0.0437
2	0.0226	-	0.0410
3	0.0437	0.0410	-

Average distances (expected heterozygosity) between individuals in same cluster:

```
cluster  1  : 0.5583
cluster  2  : 0.5809
cluster  3  : 0.5314
```

Table S8b: Detailed results from Structure analysis – populations from flowers

Proportion of membership of each pre-defined population in each of the 3 clusters:

Given Pop	Inferred Clusters 1	2	3	Number of Individuals
1:	0.231	0.371	0.398	6
2:	0.354	0.346	0.300	10
3:	0.352	0.339	0.309	6
4:	0.197	0.411	0.392	7
5:	0.303	0.335	0.361	8
6:	0.330	0.387	0.283	5
7:	0.377	0.272	0.351	10
8:	0.336	0.288	0.376	10
9:	0.443	0.246	0.311	10
10:	0.462	0.315	0.223	6
11:	0.386	0.300	0.314	6
12:	0.258	0.485	0.257	6
13:	0.191	0.307	0.502	6
14:	0.420	0.348	0.232	6
15:	0.361	0.154	0.484	6
16:	0.257	0.323	0.420	6
17:	0.337	0.520	0.143	6
18:	0.299	0.339	0.362	10
19:	0.316	0.298	0.386	8
20:	0.219	0.462	0.319	6
21:	0.228	0.334	0.438	6
22:	0.278	0.510	0.212	10
23:	0.317	0.345	0.338	6
24:	0.391	0.300	0.309	6
25:	0.226	0.547	0.227	6
26:	0.351	0.381	0.269	7
27:	0.279	0.332	0.389	6
28:	0.301	0.202	0.497	15
29:	0.483	0.216	0.301	6
30:	0.386	0.389	0.226	6
31:	0.303	0.490	0.207	6
32:	0.354	0.388	0.258	6
33:	0.311	0.367	0.323	6
34:	0.193	0.487	0.321	6
35:	0.372	0.391	0.237	6
36:	0.233	0.377	0.390	6
37:	0.262	0.404	0.333	6
38:	0.393	0.313	0.294	6
39:	0.337	0.440	0.223	6
40:	0.246	0.371	0.383	6
41:	0.334	0.325	0.341	15
42:	0.241	0.227	0.532	6
43:	0.248	0.419	0.333	6
44:	0.269	0.424	0.307	6
45:	0.250	0.339	0.411	5
46:	0.286	0.228	0.487	6
47:	0.393	0.383	0.225	6
48:	0.435	0.310	0.254	6
49:	0.362	0.323	0.315	6
50:	0.256	0.401	0.343	7
51:	0.332	0.300	0.368	10
52:	0.401	0.434	0.165	5
53:	0.252	0.392	0.356	8
54:	0.449	0.259	0.291	6
55:	0.260	0.306	0.434	6
56:	0.223	0.384	0.392	6
57:	0.258	0.486	0.255	6
58:	0.283	0.447	0.270	6
59:	0.373	0.378	0.249	6
60:	0.497	0.178	0.325	6
61:	0.259	0.366	0.375	6
62:	0.353	0.207	0.440	6
63:	0.349	0.312	0.339	6
64:	0.323	0.361	0.316	6
65:	0.294	0.287	0.419	5

66:	0.278	0.370	0.352	10
67:	0.406	0.268	0.326	6
68:	0.345	0.307	0.348	10
69:	0.449	0.264	0.287	6
70:	0.253	0.306	0.441	15
71:	0.344	0.348	0.308	6
72:	0.290	0.287	0.422	6
73:	0.310	0.380	0.311	15
74:	0.305	0.482	0.213	8
75:	0.334	0.283	0.383	10
76:	0.267	0.468	0.265	10
77:	0.243	0.313	0.444	10

Names of population-districts are in Supplement Table S1.

Allele-freq. divergence among pops (Net nucleotide distance), computed using point estimates of P.

	1	2	3
1	-	0.0404	0.0463
2	0.0404	-	0.0774
3	0.0463	0.0774	-

Average distances (expected heterozygosity) between individuals in same cluster:

```
cluster  1  : 0.6100
cluster  2  : 0.5167
cluster  3  : 0.5192
```
